# A Co-Localization Algorithm for Underwater Moving Targets with an Unknown Constant Signal Propagation Speed and Platform Errors

**DOI:** 10.3390/s24103127

**Published:** 2024-05-14

**Authors:** Yang Liu, Long He, Gang Fan, Xue Wang, Ya Zhang

**Affiliations:** 1 College of Mechatronics Engineering, North University of China, Taiyuan 030051, China; 2 School of Mathematics and Statistics, Wuhan University, Wuhan 430072, China

**Keywords:** underwater mobile target localization, AOA/TDOA/FDOA, unknown sound speed, platform systematic errors, closed-form algorithm, CRLB

## Abstract

Underwater mobile acoustic source target localization encounters several challenges, including the unknown propagation speed of the source signal, uncertainty in the observation platform’s position and velocity (i.e., platform systematic errors), and economic costs. This paper proposes a new two-step closed-form localization algorithm that jointly estimates the angle of arrival (AOA), time difference of arrival (TDOA), and frequency difference of arrival (FDOA) to address these challenges. The algorithm initially introduces auxiliary variables to construct pseudo-linear equations to obtain the initial solution. It then exploits the relationship between the unknown and auxiliary variables to derive the exact solution comprising solely the unknown variables. Both theoretical analyses and simulation experiments demonstrate that the proposed method accurately estimates the position, velocity, and speed of the sound source even with an unknown sound speed and platform systematic errors. It achieves asymptotic optimality within a reasonable error range to approach the Cramér–Rao lower bound (CRLB). Furthermore, the algorithm exhibits low complexity, reduces the number of required localization platforms, and decreases the economic costs. Additionally, the simulation experiments validate the effectiveness of the proposed localization method across various scenarios, outperforming other comparative algorithms.

## 1. Introduction

Underwater mobile acoustic source target localization technology has been a significant research challenge in marine scientific research, resource exploration, and military applications [1,2,3]. It is well known that a GNSS provides 4D positioning (XYZT) on land and water and in the air and space. GNSSs are less suitable for underwater measurements XYZT due to the known limitations of the propagation of electromagnetic waves underwater. As a rule, underwater vehicles can determine their position from XYT acoustic signals from multiple surface sources. The receiver “listens” to radio acoustic repeaters, receives their messages, and determines the geographic coordinates of the acoustic speakers [4,5]. In [6], GIBs (GPS intelligent buoys) are a group of buoys that calculate their own GPS position and then send acoustic signals to a submersible as the basis for calculating its position. Furthermore, [7] uses buoys that act as mobile pseudolites and convert the GPS radio service into a service based on underwater acoustics. Using this service, an unlimited number of users can independently determine locations and navigate underwater. Briefly, a range of acoustic detection platforms (hereafter referred to as platforms), like underwater vehicles (UUVs) [8,9,10], underwater sensor networks (USNs) [11,12,13], and surface buoys [6,7], can use their position, velocity, and other information to provide the necessary assistance in the localization of underwater acoustic sources. These multiple underwater platforms enable the acquisition and processing of underwater acoustic source signals through acoustic sensors. The parameter information extracted from these signals, including the time, frequency, spatial, and energy domains, facilitates target localization. This information includes time of arrival (TOA) [14], time difference of arrival (TDOA) [10,12,15], frequency difference of arrival (FDOA) [16], angle of arrival (AOA) [11,17], signal strength (RSS) [18], and various combinations thereof [2,3,19,20,21,22]. However, the TOA method necessitates precise clock synchronization, which is both demanding and expensive in terms of the hardware equipment. Signal strength is influenced by water temperature, salinity, and flow velocity, resulting in low positioning accuracy. Combining the AOA and TDOA can only be used to estimate the position of the target [19,21], so currently, joint TDOA/FDOA positioning is often used for mobile target localization [8,22,23], which can achieve the estimation of the position and velocity to the target. However, this method demands the participation of at least five or more platforms in the localization, significantly elevating its economic costs and deployment complexities. Integrating bearing information can enhance the localization performance and reduce the number of required platforms. Thus, in this study, we will develop algorithms jointly utilizing AOA, TDOA, and FDOA observations.

The fusion of the above observations is typically nonlinear, rendering target localization a challenging problem. Consequently, numerous localization algorithms have been proposed to address this issue. The typical algorithms encompass closed-form and non-closed-form solution approaches. The closed-form solution algorithms primarily consist of the two-step weighted least squares (TS-WLS) algorithm [1,21,24,25], weighted spherical interpolation (WSI) [26], and similar techniques. The principal non-closed solution methods include the Taylor series iterative method and the semidefinite programming algorithm (SDP) [2,3,11,22]. Closed-form solutions exhibit low computational complexity. However, in the presence of significant measurement noise, the algorithm’s localization performance may suffer. Conversely, the Taylor series iterative method demands precise initial value selection and multiple iterations. On the other hand, the SDP algorithm, while demonstrating superior performance in high-error scenarios, imposes strict relaxation requirements and entails greater computational complexity. In this paper, we opt for the TS-WLS algorithm due to its closed-form solution and low computational complexity, and improvements have been made on that basis.

**Figure 1:**
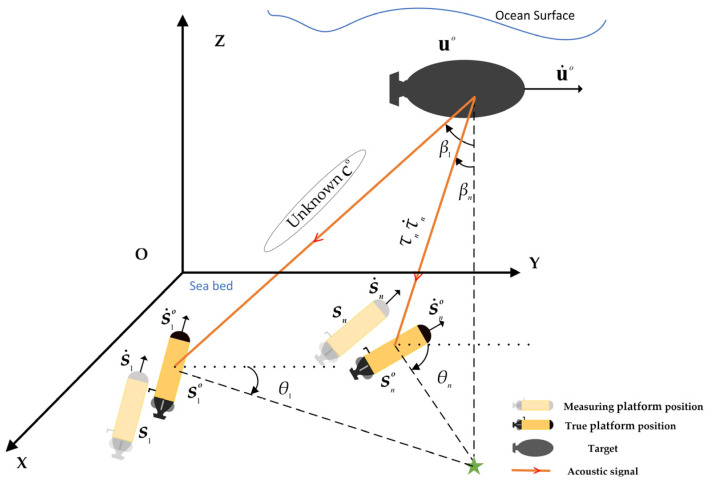
Underwater multi-platform positioning model.

The aforementioned algorithms can attain the Cramér–Rao lower bound (CRLB) under reasonable observation error conditions [27]. However, due to the complexity of the marine environment, these localization algorithms are not directly applicable to underwater multi-platform localization scenarios. Figure 1 illustrates a real underwater multi-platform localization model. Firstly, sensors typically measure the platform’s underwater position, velocity, and other parameters, which suffer from inaccuracies and are inevitably influenced by measurement errors. We refer to these inaccuracies as the platform’s systematic errors. The neglect of such errors significantly degrades the localization performance [28]. Secondly, the propagation speed of the acoustic source signal is influenced by water temperature, water pressure, and seawater salinity [29,30], introducing uncertainty. In certain shallow water areas [31], it may even be entirely unpredictable. Therefore, in this study, we treat the sound source signal velocity as an unknown constant.

Some algorithms have been proposed to jointly estimate the propagation velocity of the sound source signal and the target source location in response to these issues. However, most of these existing algorithms are inadequate. Rui and Ho [32] proposed a four-step closed-form solution algorithm for localization, capable of estimating the sound velocity and target location. However, it is limited to two-dimensional scenarios and involves three pseudo-linear transformations to derive the closed-form solution, making the process cumbersome. Yang [31] employed the semidefinite relaxation technique (SDR) to convert a nonconvex localization problem into a semidefinite programming problem (SDP) to estimate the target position and sound velocity. Although it enhances the target localization accuracy in the presence of substantial sensor measurement errors, it demands significant computational resources. Fan [33] and Jia [34] enhanced the four-step method proposed by Rui and Ho [32] using the Lagrange multiplier method and the generalized trust region subproblem (GTRS), respectively, simplifying it into a two-step process to reduce the algorithmic complexity. However, these methods necessitate clock synchronization constraints. Sun [35] considers clock synchronization errors and employs a two-step weighting algorithm to estimate the target position and velocity, thereby enhancing the localization accuracy. However, these methods are tailored to multi-base static sonar scenarios and are solely capable of estimating the target position. Zhang [36] introduces a joint localization closure solution algorithm based on TDOA-FDOA, capable of estimating the position, velocity, and speed of sound of the moving target. However, it necessitates iterative solving, thereby increasing the algorithmic complexity. Additionally, the algorithm stipulates that the number of cooperative platforms involved in localization should not be fewer than five, potentially restricting its applicability in practical underwater localization scenarios. In conclusion, the current literature lacks a joint AOA/TDOA/FDOA method to address the unknown propagation speed of sound source signals and the uncertainty in the position and velocity information on moving platforms in underwater moving sound source target research.

This paper addresses the localization of underwater moving sound source targets using multiple mobile platforms with joint AOA/TDOA/FDOA and presents a two-step closed-form localization algorithm. Additionally, we derive and analyze the corresponding CRLB, conduct algorithm performance and complexity analyses, and verify through theoretical and simulation studies that the proposed method achieves the CRLB within an acceptable error margin. The primary contributions and innovations of this paper include:

- We propose a novel joint AOA/TDOA/FDOA target localization algorithm for underwater moving sound sources. This algorithm effectively estimates the target sound source position, velocity, and sound source signal velocity even under an unknown sound velocity and platform systematic error conditions. Additionally, it reduces the number of localization platforms, thus lowering the economic costs.
- We develop a new two-step closed-form solution that introduces fewer auxiliary variables, resulting in an exact solution containing only unknown variables. This approach avoids complex iterative operations and reduces the computational complexity of the algorithm.
- The proposed algorithm demonstrates asymptotic optimality within an acceptable error range. In five distinct localization scenarios, the root mean square error of the estimated parameters reaches the CRLB.
- The proposed method achieves effective localization even in scenarios involving fewer moving platforms, uncertain platform–target geometries, and far-field acoustic source targets. It also surpasses the other compared algorithms in terms of estimation performance.

The remainder of the paper is structured as follows: Section 2 provides a detailed description of the underwater localization model utilized in this paper. Section 3 derives the CRLB for this localization model and investigates the impact of an unknown sound speed on optimal estimation through simulation experiments. Section 4 outlines the localization method adopted in this paper. Section 5 thoroughly analyzes the performance and computational complexity of the algorithms proposed in this paper. The numerical simulation experiments in the five scenarios presented in Section 6 validate the correctness of the preceding theory and demonstrate the superiority of the algorithm proposed in this paper over other algorithms. Section 7 provides a summary of the entire paper.

The following notation will be used throughout this paper. Bold lowercase letters such as s denote vectors, bold uppercase letters such as S denote matrices, $S^{T}$ denotes the transpose matrix of S, ${\left(•\right)}^{o}$ denotes the actual value of (•), ∥a∥ denotes the 2-norm of the vector a, $O_{m\times n}$ denotes the zero matrix of m×n, $I_{m\times m}$ denotes the unit matrix of m×m, ⊗ is the Kronecker product, diag(a,b) is a diagonal matrix with the diagonal elements a,b, $\mathrm{blakdiag}\left[R_{1},R_{2}\right]$ is a matrix with $R_{1}$ and $R_{2}$ on the diagonal and 0 as the other elements, A(n,:) denotes the $n_{th}$ row vector of the matrix A, $G\left(n,j:k\right)$ is a vector consisting of the $n_{th}$ row and the $j_{th}$ to $k_{th}$ column elements of the matrix G, and Ψ(j:k) is a vector consisting of the $j_{th}$ to $k_{th}$ elements of the vector Ψ.

## 2. Localization Model

This section describes the utilization of a multi-system comprising N underwater mobile platforms to localize a single moving sound source target. As depicted in Figure 1, first, we define the true position of the moving target source: $u^{o}={\left[\begin{array}{ccc} x & y & z \end{array}\right]}^{T}$, ${\dot{u}}^{o}={\left[\begin{array}{ccc} \dot{x} & \dot{y} & \dot{z} \end{array}\right]}^{T}$. The true position and true velocity of the moving platform are, respectively, $s^{o}={\left[{\left(s_{1}^{o}\right)}^{T},{\left(s_{2}^{o}\right)}^{T},\ldots ,{\left(s_{N}^{o}\right)}^{T}\right]}^{T}$, ${\dot{s}}^{o}={\left[{\left({\dot{s}}_{1}^{o}\right)}^{T},{\left({\dot{s}}_{2}^{o}\right)}^{T},\ldots ,{\left({\dot{s}}_{N}^{o}\right)}^{T}\right]}^{T}$, where $s_{n}^{o}={\left[x_{n}^{o}\;y_{n}^{o}{\;z}_{n}^{o}\right]}^{T}$, ${\dot{s}}_{n}^{o}={\left[{\dot{x}}_{n}^{o}\;{\dot{y}}_{n}^{o}{\dot{z}}_{n}^{o}\right]}^{T}$, n=1,2,…,N. In practice, the position and speed of underwater mobile platforms are also measured using sensors, so they will be affected by measurement errors, which are hereinafter collectively referred to as mobile platform systematic errors. Denoting $s_{n}=s_{n}^{o}+\Delta s_{n}$ and ${\dot{s}}_{n}={\dot{s}}_{n}^{o}+\Delta {\dot{s}}_{n}$, $\Delta s_{n}$ and $\Delta {\dot{s}}_{n}$ are random measurement errors. The position and velocity parameters of each moving platform can be represented in vector form.

(1) $$S=S^{o}+\Delta S.$$

In (1), $S={\left[s^{T},{\dot{s}}^{T}\right]}^{T}={\left[{\left(s_{1}\right)}^{T},{\left(s_{2}\right)}^{T},\ldots ,{\left(s_{N}\right)}^{T},{\left({\dot{s}}_{1}\right)}^{T},{\left({\dot{s}}_{2}\right)}^{T},\ldots ,{\left({\dot{s}}_{N}\right)}^{T}\right]}^{T}$ and $S^{o}={\left[s^{oT},{\dot{s}}^{oT}\right]}^{T}$ are used to denote the vector of the measured parameters of the moving platforms and the vector of the actual parameters, $\Delta S={\left[\Delta s^{T},\Delta {\dot{s}}^{T}\right]}^{T}={\left[{\left(\Delta s_{1}\right)}^{T},{\left(\Delta s_{2}\right)}^{T},\ldots ,{\left(\Delta s_{N}\right)}^{T},{\left(\Delta {\dot{s}}_{1}\right)}^{T},{\left(\Delta {\dot{s}}_{2}\right)}^{T},\ldots ,{\left(\Delta {\dot{s}}_{N}\right)}^{T}\right]}^{T}$, ΔS follows a Gaussian distribution with a mean of zero. Its covariance matrix is $E(\Delta S^{T}\Delta S)=Q_{S}$.

Figure 1 illustrates that the platform can measure the azimuth angle of the target as $\theta_{n}=\theta_{n}^{o}+\Delta \theta_{n}$ and the pitch angle as $\beta_{n}{=\beta }_{n}^{o}+\Delta \beta_{n}$, where $\theta_{n}^{o}$ and $\beta_{n}^{o}$ denote the true angle; the precise observation equation for the AOA obtained by platform n is

(2) $$\begin{array}{l} \theta_{n}^{o}=\arctan\left(\left(y-y_{n}^{o}\right)/\left(x-x_{n}^{o}\right)\right),\\ \beta_{n}^{o}=\arctan\left((z-z_{n}^{o})/\sqrt{{\left(x-x_{n}^{o}\right)}^{2}+{\left(y-y_{n}^{o}\right)}^{2}}\right). \end{array}$$

We can represent the angle information measured by each moving platform in vector form

(3) $$\theta =\theta^{o}+\Delta \theta ,\beta =\beta^{o}+\Delta \beta .$$

In (3), $\theta ={\left[\theta_{1},\theta_{2},\ldots ,\theta_{N}\right]}^{T}$ and $\beta ={\left[\beta_{1},\beta_{2},\cdots ,\beta_{N}\right]}^{T}$ denote the measured angle vectors, $\theta^{o}=[\theta_{1}^{o},\theta_{2}^{o},\ldots \theta_{N}^{o}]$ and $\beta^{o}={\left[\beta_{1}^{o},\beta_{2}^{o},\cdots ,\beta_{N}^{o}\right]}^{T}$ denote the true angle vectors, and $\Delta \theta ={\left[\Delta \theta_{1},\Delta \theta_{2},\cdots ,\Delta \theta_{N}\right]}^{T}$ and $\Delta \beta ={\left[\Delta \beta_{1},\Delta \beta_{2},\cdots ,\Delta \beta_{N}\right]}^{T}$ conform to a Gaussian distribution with a mean of zero. Their covariance matrices are, respectively, $E(\Delta \theta^{T}\Delta \theta )=Q_{\theta }$ and $E(\Delta \beta^{T}\Delta \beta )=Q_{\beta }$.

Using the first moving platform as the reference point, the exact observation equation for the TDOA obtained for the nth platform is

(4) $$\tau_{n}^{o}=\frac{1}{c^{o}}(R_{n}^{o}-R_{1}^{o})=\frac{1}{c^{o}}\left(∥u^{o}-s_{n}^{o}∥-∥u^{o}-s_{1}^{o}∥\right),n=2,3,\ldots ,N,$$

where $R_{n}^{o}=∥u^{o}-s_{n}^{o}∥$ denotes the true distance from the $n_{\mathrm{th}}$ platform to the target.

Delay measurements typically incur errors. We denote $\tau_{n}=\tau_{n}^{o}+\Delta \tau_{n}$. Measurements from multiple platforms can be represented in vector form, as

(5) $$\tau =\tau^{o}+\Delta \tau .$$

In (5), $\tau ={\left[\tau_{2},\tau_{3},\cdots ,\tau_{N}\right]}^{T}$,$\tau^{o}={\left[\tau_{2}^{o},\tau_{3}^{o},\cdots ,\tau_{N}^{o}\right]}^{T}$, respectively, represent the measured delay parameter vector and the actual parameter vector. $\Delta \tau ={\left[\Delta \tau_{2},\Delta \tau_{3},\cdots ,\Delta \tau_{N}\right]}^{T}$, Δτ is a Gaussian error vector with a mean of zero, and its covariance matrix is $E(\Delta \tau^{T}\Delta \tau )=Q_{\tau }$.

Deriving the observation equation for the TDOA yields the exact observation equation for the FDOA.

(6) $${\dot{\tau }}_{n}^{o}=\frac{1}{c^{o}}({\dot{R}}_{n}^{o}-{\dot{R}}_{1}^{o}),n=2,3,\ldots ,N,$$

where ${\dot{R}}_{n}^{o}=\frac{{\left(u^{o}-s_{n}^{o}\right)}^{T}\left({\dot{u}}^{o}-{\dot{s}}_{n}^{o}\right)}{R_{n}^{o}}$ represents the rate of the change in the radial distance difference between the $n_{\mathrm{th}}$ platform and the target.

Frequency difference measurements typically contain errors. We denote ${\dot{\tau }}_{n}={\dot{\tau }}_{n}^{o}+\Delta {\dot{\tau }}_{n}$; the frequency difference measurements from multiple platforms can be represented in vector form

(7) $$\dot{\tau }={\dot{\tau }}^{o}+\Delta \dot{\tau }.$$

In (7), $\dot{\tau }={\left[{\dot{\tau }}_{2},{\dot{\tau }}_{3},\cdots ,{\dot{\tau }}_{N}\right]}^{T}$ and $\tau^{o}={\left[{\dot{\tau }}_{2}^{o},{\dot{\tau }}_{3}^{o},\cdots ,{\dot{\tau }}_{N}^{o}\right]}^{T}$, respectively, represent the measured frequency difference parameter vector and the actual frequency difference parameter vector, $\Delta \dot{\tau }={\left[\Delta {\dot{\tau }}_{2},\Delta {\dot{\tau }}_{3},\cdots ,\Delta {\dot{\tau }}_{N}\right]}^{T}$, $\Delta \dot{\tau }$ represents the Gaussian distribution delay error vector with a mean of zero, and its covariance matrix is $E(\Delta {\dot{\tau }}^{T}\Delta \dot{\tau })=Q_{\dot{\tau }}$.

Combining the measurements of the AOA, TDOA, and FDOA and integrating them into an (4N−2) matrix, we obtain

(8) $$\gamma ={\left[\theta^{T},\beta^{T},\tau^{T},{\dot{\tau }}^{T}\right]}^{T}=\gamma^{o}+\Delta \gamma ,$$

(9) $$Q_{\gamma }=E(\Delta \gamma^{T}\Delta \gamma )=\mathrm{bladiag}\left(Q_{\theta },Q_{\beta },Q_{\tau },Q_{\dot{\tau }}\right),$$

where $\gamma^{o}={\left[\theta^{oT},\beta^{oT},\tau^{oT},{\dot{\tau }}^{oT}\right]}^{T}$,$\Delta \gamma ={\left[\Delta \theta^{T},\Delta \beta^{T},\Delta \tau^{T},\Delta {\dot{\tau }}^{T}\right]}^{T}$, and $Q_{\gamma }$ is the measurement error covariance matrix.

## 3. Cramér–Rao Lower Bound Analysis

The CRLB reflects the optimal statistical performance obtained using all the unbiased estimates. Therefore, before presenting the proposed algorithm, this section first provides the CRLB of the localization model described in Section 2 as a standard for evaluating the estimation performance. And through simulation experiments, we verify the impact of an unknown sound speed and the uncertainty of the positions and velocities of the mobile platforms on the optimal estimation accuracy.

### 3.1. Derivation of the CRLB

The unknown vector is represented as $\xi ={\left[S^{oT},c^{o},\varphi^{oT}\right]}^{T}$, where $S^{o}$ is a random parameter, $\varphi^{o}={\left[u^{T},{\dot{u}}^{T}\right]}^{T}$, and $c^{o}$ represents the unknown parameters. By combining the observation vector $\gamma ={\left[\theta^{T},\beta^{T},\tau^{T},{\dot{\tau }}^{T}\right]}^{T}$ with the platform parameter vector S containing systematic errors, we can obtain the log-likelihood function of the joint probability density function of the target state estimates under the Gaussian data model as

(10) $$\ln P\left(\gamma ,S;\xi \right)=F-\frac{1}{2}{\left(\gamma -\gamma^{o}\right)}^{T}Q_{\gamma }^{-1}\left(\gamma -\gamma^{o}\right)-\frac{1}{2}{\left(S^{o}-S\right)}^{T}Q_{S}^{-1}\left(S^{o}-S\right),$$

where $F=-0.5\ln({\left(2\pi \right)}^{4\times N}\left|Q_{\gamma }\right|)-0.5\ln({\left(2\pi \right)}^{6\times N}\left|Q_{s}\right|)$, and F is a constant term independent of ξ.

According to the estimation theory [27], the CRLB estimate with respect to the unknown vector ξ is obtained by taking the second-order derivative of the likelihood function with respect to ξ and taking the inverse of the expectation matrix, which partitions the CRLB into a 3 × 3 block matrix, namely

(11) $$\mathrm{CLRB}\left(\xi \right)=-E{\left[\frac{\partial^{2}\ln P\left(\gamma ;\xi \right)}{\partial \xi \partial \xi^{T}}\right]}^{-1}={\left[\begin{array}{ccc} X_{11} & X_{12} & X_{13}\\ {X_{12}}^{T} & X_{22} & X_{23}\\ {X_{13}}^{T} & {X_{23}}^{T} & X_{33} \end{array}\right]}^{-1}.$$

From (11), each block matrix corresponds to the estimated performance of $\varphi^{o},S^{o}\;\mathrm{and}{\;c}^{o}$, and their expressions are

(12) $$\begin{array}{l} X_{11}=-E\left[\frac{\partial^{2}\ln P\left(\gamma ;\xi \right)}{\partial \varphi^{o}\partial \varphi^{oT}}\right]={\left(\frac{\partial \gamma^{o}}{\partial \varphi^{o}}\right)}^{T}Q_{\gamma }^{-1}\left(\frac{\partial \gamma^{o}}{\partial \varphi^{o}}\right),X_{12}=-E\left[\frac{\partial^{2}\ln P\left(\gamma ;\xi \right)}{\partial \varphi^{o}\partial c^{o}}\right]={\left(\frac{\partial \gamma^{o}}{\partial \varphi^{o}}\right)}^{T}Q_{\gamma }^{-1}\left(\frac{\partial \gamma^{o}}{\partial c^{o}}\right),\\ X_{22}=-E\left[\frac{\partial^{2}\ln P\left(\gamma ;\xi \right)}{\partial c^{o}\partial c^{o}}\right]={\left(\frac{\partial \gamma^{o}}{\partial c^{o}}\right)}^{T}Q_{\gamma }^{-1}\left(\frac{\partial \gamma^{o}}{\partial c^{o}}\right),X_{13}=-E\left[\frac{\partial^{2}\ln P\left(\gamma ;\xi \right)}{\partial \varphi^{o}\partial {\left(S^{o}\right)}^{T}}\right]={\left(\frac{\partial \gamma^{o}}{\partial \varphi^{o}}\right)}^{T}Q_{\gamma }^{-1}\left(\frac{\partial \gamma^{o}}{\partial S^{o}}\right),\\ X_{23}=-E\left[\frac{\partial^{2}\ln P\left(\gamma ;\xi \right)}{\partial c^{o}\partial {\left(S^{o}\right)}^{T}}\right]={\left(\frac{\partial \gamma^{o}}{\partial c^{o}}\right)}^{T}Q_{\gamma }^{-1}\left(\frac{\partial \gamma^{o}}{\partial S^{o}}\right),X_{33}=-E\left[\frac{\partial^{2}\ln P\left(\gamma ;\xi \right)}{\partial S^{o}\partial {\left(S^{o}\right)}^{T}}\right]={\left(\frac{\partial \gamma^{o}}{\partial S^{o}}\right)}^{T}Q_{\gamma }^{-1}\left(\frac{\partial \gamma^{o}}{\partial S^{o}}\right)+Q_{S}^{-1}. \end{array}$$

A more detailed derivation of the expression of each partial derivative in (12) is provided in Appendix A.

Next, we examine how the speed of sound propagation and perturbations in the position and velocity of the moving platform affect the localization performance. To emphasize the impact of two conditions—the unknown speed of sound and the presence of systematic errors—on the localization performance, we explore the CRLB with and without systematic errors and with knowledge of the unknown speed of sound. We examine this under two scenarios.

1. $c^{o}$ is known: When the speed of sound is known, the partial derivative of (11) with respect to $c^{o}$ is zero, as follows:

(13) $$\mathrm{CRLB}\left(\varphi^{o}\right)={\left[\begin{array}{cc} X_{11} & X_{13}\\ {X_{13}}^{T} & X_{33} \end{array}\right]}^{-1}.$$

Using our knowledge of matrix chunking theory [37], the CRLB of the unknown vector $\varphi^{o}$ can be obtained as:

(14) $$\begin{array}{ll} \mathrm{CLRB}\left(\varphi^{o}\right) & ={\left(X_{11}-X_{13}X_{33}^{-1}X_{13}^{T}\right)}^{-1}\\ & ={X_{11}}^{-1}+{X_{11}}^{-1}X_{13}{\left(X_{33}-X_{13}^{T}{X_{11}}^{-1}X_{13}\right)}^{-1}X_{13}^{T}{X_{11}}^{-1}. \end{array}$$

In (14), ${X_{11}}^{-1}$ represents the CRLB when the speed of sound is known and there are no systematic errors, and the second term on the right-hand side indicates the performance loss due to the presence of systematic errors.

2. $c^{o}$ is unknown: When the speed of sound is completely unknown, it is possible to jointly estimate the unknown information on the speed of sound and the observed target, and we denote $\nu^{o}={\left[\varphi^{oT},c^{o}\right]}^{T}$, obtaining the CRLB of $\nu^{o}$ as

(15) $$\begin{array}{ll} \mathrm{CRLB}(\nu^{o}) & ={\left(\left[\begin{array}{ll} X_{11} & X_{12}\\ X_{12}^{T} & X_{22} \end{array}\right]-\left[X_{13}X_{23}\right]X_{33}^{-1}\left[\begin{array}{l} X_{13}^{T}\\ X_{23}^{T} \end{array}\right]\right)}^{-1}\\ & ={\left(X_{c}-X_{d}X_{33}^{-1}X_{d}^{T}\right)}^{-1}={X_{c}}^{-1}+{X_{c}}^{-1}X_{d}{\left(X_{33}-X_{d}^{T}{X_{c}}^{-1}X_{d}\right)}^{-1}X_{d}^{T}{X_{c}}^{-1}, \end{array}$$

where $X_{c}={\left[\frac{\partial \gamma^{o}}{\partial \varphi }\;\frac{\partial \gamma^{o}}{\partial c^{o}}\right]}^{T}Q_{\gamma }^{-1}\left[\frac{\partial \gamma^{o}}{\partial \varphi }\;\frac{\partial \gamma^{o}}{\partial c^{o}}\right]$, $X_{d}={\left[\frac{\partial \gamma^{o}}{\partial \varphi }\;\frac{\partial \gamma^{o}}{\partial c^{o}}\right]}^{T}Q_{\gamma }^{-1}\frac{\partial \gamma^{o}}{\partial S^{o}}$.

The first term on the right-hand side of (15) incorporates the effect of the unknown acoustic propagation velocity on the CRLB of φ. The second term then contains the effect of the unknown acoustic signal propagation velocity and the platform system errors on the CRLB of $\nu^{o}$, which is clearly not generated in an additive manner. If the effect of systematic errors is not considered, i.e., $\frac{\partial \gamma^{o}}{\partial S^{o}}=0$, then the first term on the right-hand side of (15) is the CRLB estimate of $\nu^{o}$. At this point, the CRLB values of the unknown vectors $\varphi^{o}$ and $c^{o}$ are

(16) $$\left\{\begin{array}{l} \mathrm{CLRB}\left(\varphi^{o}\right)={X_{11}}^{-1}+{X_{11}}^{-1}X_{12}\mathrm{CLRB}\left(c^{o}\right)X_{12}^{T}{X_{11}}^{-1}\\ \mathrm{CLRB}\left(c^{o}\right)={\left(X_{22}-X_{12}^{T}{X_{11}}^{-1}X_{12}\right)}^{-1} \end{array}\right..$$

The ultimate CRLB estimates for the unknown vectors $u^{o},{\dot{u}}^{o}\;\mathrm{and}{\;c}^{o}$ are derived as follows

(17) $${\mathrm{CRLB}}_{u}=\overset{3}{\underset{k=1}{\sum }}\mathrm{CLRB}{\left(\nu^{o}\right)}_{k,k},{\mathrm{CRLB}}_{\dot{u}}=\overset{6}{\underset{k=4}{\sum }}\mathrm{CLRB}{\left(\nu^{o}\right)}_{k,k},{\mathrm{CRLB}}_{c^{o}}=\mathrm{CLRB}{\left(\nu^{o}\right)}_{7,7}.$$

### 3.2. Comparison of CRLB Simulation Experiments

**Figure 2:**
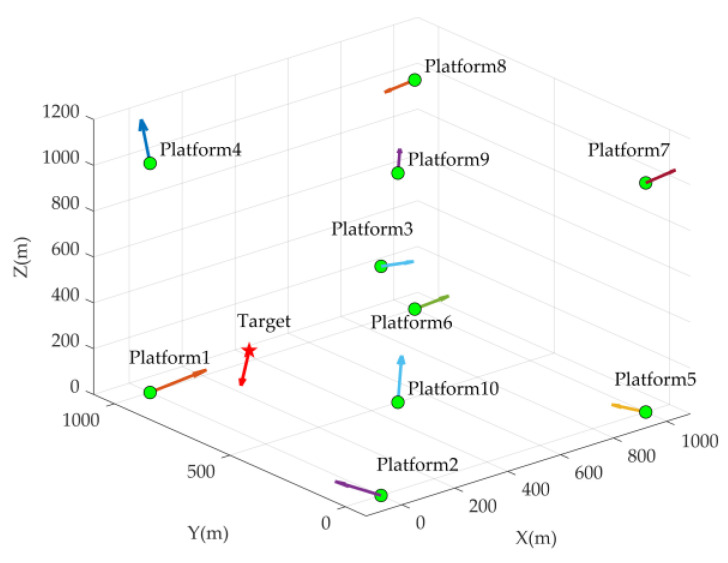
Underwater positioning scenario diagram.

To analyze the impact of the known/unknown $c^{o}$ and the systematic errors of the mobile platform on the CRLB, we conduct an analysis here by adjusting various sensor measurement errors and mobile platform systematic errors. The simulation utilizes the positioning system described in reference [36], comprising underwater mobile platforms, to estimate the position, velocity, and unknown speed of sound for the underwater targets, as illustrated in Figure 2. Here, with N=10, the actual position and velocity parameters of the platform are presented in Table 1.

**Figure 3:**
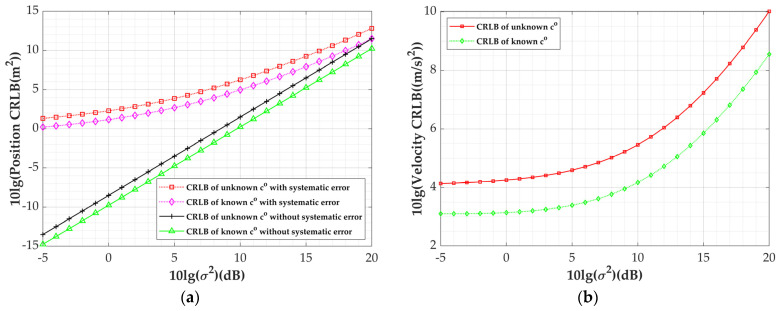
Variation in CRLBs with sensor measurement errors under unknown/known $c^{o}$: (**a**) position CRLBs; (**b**) velocity CRLBs.

**Figure 4:**
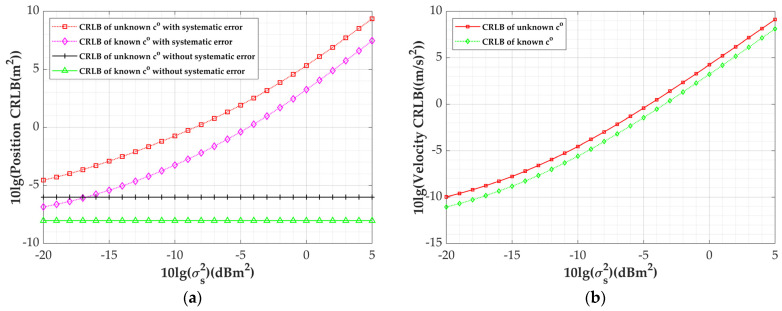
Variation in CRLBs with platform systematic errors under unknown/known $c^{o}$: (**a**) position CRLBs; (**b**) velocity CRLBs.

The simulation results are presented in Figure 3 and Figure 4. Figure 3a,b depict the variation in the CRLB for the target’s position and velocity, respectively, with sensor measurement errors under $c^{o}$ unknown/known conditions. Setting the magnitude of the systematic errors to $\sigma_{s}=1\;m$, Figure 3a also illustrates the change in the CRLB of the target’s position with sensor measurement errors when systematic errors are absent. Regardless of the presence of systematic errors, the CRLB at an unknown sound speed is higher than that with the speed of sound known. For instance, when the perturbation parameter of the observables σ=1, i.e., $10\mathrm{lg}\sigma^{2}=0$, $\sigma_{\mathrm{aoa}}=0.1\;\deg$, $\sigma_{d}=1\;m$, a difference of 1.12 dB is observed between the two in terms of the position estimation performance and 1.11 dB in terms of the speed estimation performance in the presence of systematic errors. In the absence of systematic errors, there is also a difference of 1.25 dB in the position estimation performance. Additionally, it can be observed from Figure 3a that the presence of systematic errors also diminishes the target localization accuracy when the sound speed is known/unknown.

Figure 4 shows the variation in the CRLBs incorporating the systematic error of the moving platform. The measurement error variance $\sigma_{\mathrm{aoa}}=1\;\deg$, $\sigma_{d}=1\;m$; again, it can be seen that the CRLB at an unknown sound speed is higher than the CRLB with the speed of sound known. It can also be seen from Figure 4b that when the systematic error is less than −17 dB, the CRLB with systematic error and with the speed of sound known is higher than the position estimation error at an unknown sound speed and with no systematic error. This indicates that the smaller the error, the greater the effect of the speed of sound being unknown on localization.

**Figure 5:**
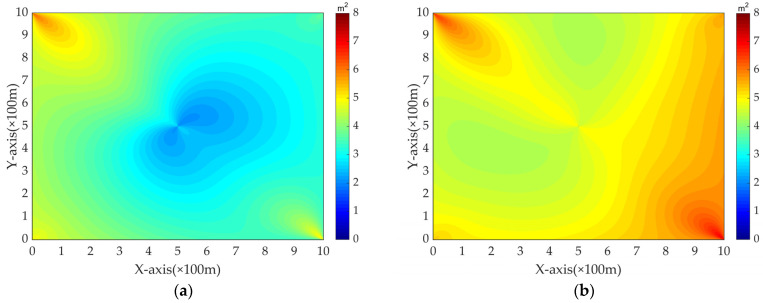
CRLBs for moving targets at different positions: (**a**) CRLBs for target position with known $c^{o}$; (**b**) CRLBs for target position with unknown $c^{o}$.

In order to better verify the localization performance of moving targets when the speed of sound is unknown/known, it is assumed that the parameters of the underwater mobile observation platform are the same as above, $\sigma_{\mathrm{aoa}}=1\;\deg$, $\sigma_{d}=1\;m$, $\sigma_{s}=1\;m$. The unknown moving target moves from a water area of ${\left[0,0,200\right]}^{T}m$ from left to right at a speed of 1 m/s until it reaches a water area of ${\left[1000,1000,200\right]}^{T}m$ with a localization period of 1 s. Figure 5 gives the CRLBs in the two cases with the moving targets at different positions. It can be seen that at the same position, the CRLB values in Figure 5b are generally larger than the CRLB values corresponding to Figure 5a.

In summary, the simulation also verifies the previous statement that the value of the estimated CRLB for the position and velocity of the target increases when the speed of sound is unknown and there is uncertainty in the information on the position and velocity of the moving platform. Therefore, it is necessary for us to design a joint AOA/TDOA/FDOA-based localization algorithm to achieve an asymptotically optimal statistical performance under these two conditions.

## 4. Positioning Algorithms

In this section, we will discuss the underwater mobile target localization problem with inaccurate mobile platform position and velocity information and completely unknown sound source signal velocity information. We propose a two-step closed-form localization algorithm. In the first stage, we minimize the introduction of additional unknowns to formulate pseudo-linear equations. After linearization, we obtain an initial solution. In the second stage, we derive an exact solution containing only unknown variables by considering the relationship between the source parameters and redundant variables. The specific process will be described below.

### 4.1. The First-Step Calculation Principle

First, we need to linearize the three joint localization equations so that we can construct a linear data model and thus roughly estimate the estimated target position $u^{o}$, velocity ${\dot{u}}^{o}$, and speed of sound $c^{o}$.

1. For AOA: First, (2) can be transformed into a pseudo-linear equation

(18) $$\left\{\begin{array}{l} \left(x-x_{n}^{o}\right)\sin\left(\theta_{n}^{o}\right)-\left(y-y_{n}^{o}\right)\cos\left(\theta_{n}^{o}\right)=0\Rightarrow \omega_{1}^{T}\left(\theta_{n}^{o}\right)u=\omega_{1}^{T}\left(\theta_{n}^{o}\right)s_{n}^{o},\\ =\left(\left(x-x_{n}^{o}\right)\cos\left(\theta_{n}^{o}\right)+\left(y-y_{n}^{o}\right)\sin\left(\theta_{n}^{o}\right)\right)\sin\left(\beta_{n}^{o}\right)-\left(z-z_{n}^{o}\right)\cos\left(\beta_{n}^{o}\right)=0\\ \Rightarrow \omega_{2}^{Τ}\left(\theta_{n}^{o},\beta_{n}^{o}\right)u=\omega_{2}^{T}\left(\theta_{n},\beta_{n}^{o}\right)s_{n}^{o}, \end{array}\right.$$

where $\omega_{1}\left(\theta_{n}^{o}\right)={\left[-\sin\left(\theta_{n}^{o}\right),\cos\left(\theta_{n}^{o}\right),0\right]}^{T}$, $\omega_{2}\left(\theta_{n}^{o},\beta_{n}^{o}\right)=\left[-\sin\left(\beta_{n}^{o}\right)\cos\left(\theta_{n}^{o}\right),\right.$ ${\left.-\sin\left(\beta_{n}^{o}\right)\sin\left(\theta_{n}^{o}\right),\cos\left(\beta_{n}^{o}\right)\right]}^{T}$.

The first-order Taylor expansion of $\sin\left(\theta_{n}^{o}\right)$ and $\cos\left(\theta_{n}^{o}\right)$ at $\theta_{n}$ and $\sin\left(\beta_{n}^{o}\right)$ and $\cos\left(\beta_{n}^{o}\right)$ at $\beta_{n}$, respectively, leads to the following equation

(19) $$\left\{\begin{array}{l} \sin\left(\theta_{n}^{o}\right)\approx \sin\left(\theta_{n}\right)-\cos\left(\theta_{n}\right)\Delta \theta_{n},\cos\left(\theta_{n}^{o}\right)\approx \cos\left(\theta_{n}\right)+\sin\left(\theta_{n}\right)\Delta \theta_{n}\\ \sin\left(\beta_{n}^{o}\right)\approx \sin\left(\beta_{n}\right)-\cos\left(\beta_{n}\right)\Delta \beta_{n},\cos\left(\beta_{n}^{o}\right)\approx \cos\left(\beta_{n}\right)+\sin\left(\beta_{n}\right)\Delta \theta_{n} \end{array}\right..$$

Substituting (19) into (18) and performing a simple multiplicative transformation yields

(20) $$\begin{array}{l} \omega_{1}{\left(\theta_{n}\right)}^{T}s_{n}-\omega_{1}{\left(\theta_{n}\right)}^{T}u=R_{n}\cos(\beta_{n})\Delta \theta_{n}+\omega_{1}{\left(\theta_{n}\right)}^{T}\Delta s_{n},\\ {\omega_{2}}^{T}\left(\theta_{n},\beta_{n}\right)s_{n}-{\omega_{2}}^{T}\left(\theta_{n},\beta_{n}\right)u=R_{n}\Delta \beta_{n}+{\omega_{2}}^{T}\left(\theta_{n},\beta_{n}\right)\Delta s_{n}. \end{array}$$

2. For TDOA: Instead of (4), we use the available moving platform position $s_{n}$, which can be obtained by performing a first-order Taylor expansion of $∥u^{o}-s_{n}^{o}∥$ and $∥u^{o}-s_{1}^{o}∥$ at $s_{n}$ and $s_{1}$, respectively:

(21) $$\begin{array}{l} ∥u^{o}-s_{n}^{o}∥\approx ∥u^{o}-s_{n}∥+\rho_{u^{o},s_{n}}^{T}\Delta s_{n},\\ ∥u^{o}-s_{1}^{o}∥\approx ∥u^{o}-s_{1}∥+\rho_{u^{o},s_{1}}^{T}\Delta s_{1}. \end{array}$$

where $\rho_{a,b}=(a-b)/\|a-b\|$.

Substituting (21) into (4), the TDOA observation equation can be expressed as

(22) $$c^{o}\tau_{n}=∥u^{o}-s_{n}∥-∥u^{o}-s_{1}∥+\varepsilon ,$$

where $\varepsilon =\rho_{u^{o},s_{n}}^{T}\Delta s_{n}-\rho_{u^{o},s_{1}}^{T}\Delta s_{1}+c^{o}\Delta \tau_{n}$. Moving $∥u^{o}-s_{1}∥$ from the right-hand side of (22) to the left side and squaring both sides simultaneously while neglecting second-order and higher-order error terms yields the following TDOA equation.

(23) $$s_{1}^{T}s_{1}-s_{n}^{T}s_{n}+2{\left(s_{n}-s_{1}\right)}^{T}u^{o}+\tau_{n}^{2}c^{o2}+2\tau_{n}R_{1}c^{o}=2∥u^{o}-s_{n}∥\varepsilon .$$

The simplification can be obtained

(24) $$s_{1}^{T}s_{1}-s_{n}^{T}s_{n}+2{\left(s_{n}-s_{1}\right)}^{T}u^{o}+\tau_{n}^{2}c^{o2}+2\tau_{n}R_{1}c^{o}=2{\left(u^{o}-s_{n}\right)}^{T}\Delta s_{n}+2R_{n}c^{o}\Delta \tau -2\frac{R_{n}}{R_{1}}{\left(u^{o}-s_{1}\right)}^{T}\Delta s_{1}.$$

3. For FDOA: Taylor expansion of ${\dot{R}}_{1}^{o}={\left(u^{o}-s_{1}^{o}\right)}^{T}\left({\dot{u}}^{o}-{\dot{s}}_{1}^{o}\right)/R_{1}^{o}$ at $s_{1}$ and ${\dot{s}}_{1}$ can be obtained

(25) $${\dot{R}}_{1}^{o}\approx {\dot{R}}_{1}+q_{u^{o},s_{1}}^{T}\Delta s_{1}+\rho_{u^{o},s_{1}}^{T}\Delta {\dot{s}}_{1},$$

where ${\dot{R}}_{1}=\left(\frac{(u^{o}-s_{1}){\left({\dot{u}}^{o}-{\dot{s}}_{1}\right)}^{T}}{\left\|u^{o}-s_{1}\right\|}\right)$, $q_{u^{o},s_{1}}=\left(I_{3}-\left(\frac{(u^{o}-s_{1}){\left(u^{o}-s_{1}\right)}^{T}}{\|u^{o}-s_{1}{\|}^{2}}\right)\right)\frac{\left({\dot{u}}^{o}-{\dot{s}}_{1}\right)}{\left\|u^{o}-s_{1}\right\|}$.

Then, we move $R_{1}^{o}$ and ${\dot{R}}_{1}^{o}$ to the left-hand side of Equations (4) and (6), respectively, to obtain

(26) $$c\tau_{n}^{o}+R_{1}^{o}=R_{i}^{o},c{\dot{\tau }}_{n}^{o}+{\dot{R}}_{1}^{o}={\dot{R}}_{i}^{o}.$$

Multiplying both sides of Equation (26) separately leads to the transformed FDOA observation equation system as

(27) $$c^{o2}\tau_{n}^{o}{\dot{\tau }}_{n}^{o}+c^{o}{\dot{\tau }}_{n}^{o}R_{1}^{o}+c^{o}\tau_{n}^{o}{\dot{R}}_{1}^{o}={\dot{R}}_{i}^{o}R_{i}^{o}-{\dot{R}}_{1}^{o}R_{1}^{o}.$$

We substitute $\tau_{n}=\tau_{n}^{o}+\Delta \tau_{n}$, ${\dot{\tau }}_{n}={\dot{\tau }}_{n}^{o}+\Delta {\dot{\tau }}_{n}$, ${\dot{s}}_{n}={\dot{s}}_{n}^{o}+\Delta {\dot{s}}_{n}$, $s_{n}=s_{n}^{o}+\Delta s_{n}$ along with (21) and (25) into Equation (27) and neglect second-order errors to obtain

(28) $$\begin{array}{l} c^{o}R_{n}\Delta {\dot{\tau }}_{n}+c^{o}{\dot{R}}_{n}\Delta \tau_{n}+{\left({\dot{u}}^{o}-{\dot{s}}_{n}\right)}^{T}\Delta s_{n}-{\left({\dot{u}}^{o}-{\dot{s}}_{1}+c^{o}\tau_{n}q_{u^{o},s_{1}}+c^{o}{\dot{\tau }}_{n}\rho_{u^{o},s_{1}}\right)}^{T}\Delta s_{1}\\ +{\left(u^{o}-s_{n}\right)}^{T}\Delta {\dot{s}}_{n}-{\left(u^{o}-s_{1}+c^{o}\tau_{n}\rho_{u^{o},s_{1}}\right)}^{T}\Delta {\dot{s}}_{1}\\ \approx s_{1}^{T}{\dot{s}}_{1}-s_{n}^{T}{\dot{s}}_{n}+{\left({\dot{s}}_{n}-{\dot{s}}_{1}\right)}^{T}u^{o}+{\left(s_{n}-s_{1}\right)}^{T}{\dot{u}}^{o}+\tau_{n}{\dot{\tau }}_{n}c^{o2}+{\dot{\tau }}_{n}R_{1}c^{o}+\tau_{n}{\dot{R}}_{1}c^{o}. \end{array}$$

Please note that it is widely assumed in the literature that neglecting second-order compound error terms is effective under small error conditions and has been confirmed in references [31,32,33,35,36].

4. Fusion and pseudo-linear transformation of AOA/FDOA/TDOA measurements: First, collect the unknown target source parameters and two introduced auxiliary variable parameters into a vector $\psi_{1}^{o}$, where $\psi_{1}^{o}=\left[u^{o},{\dot{u}}^{o},c^{o2},R_{1}c^{o},{\dot{R}}_{1}c^{o}\right]$; then, establish a pseudo-linear equation based on this parameter vector.

First, representing Equations (20), (24), and (28) in matrix form, we have

(29) $$h_{1}-A_{1}\psi_{1}^{o}=B_{1}\Delta \gamma +D_{1}\Delta S.$$

In (29), $h_{1}=[h_{11}^{T},h_{12}^{T},h_{13}^{T},h_{14}^{T}]$ represents a (4N−2)×1 observation vector, $A_{1}={\left[A_{11}^{T},A_{12}^{T},A_{13}^{T},A_{14}^{T}\right]}^{T}$ represents a (4N−2)×9 observation vector coefficient matrix, and $D_{1}={\left[D_{11}^{T},D_{12}^{T},D_{13}^{T},D_{14}^{T}\right]}^{T}$ represents a (4N−2)×6N mobile platform system error coefficient matrix.

where $h_{11}$ and $h_{12}$ are an N×1 matrix, $A_{11}$ and $A_{12}$ are an N×9 matrix, and $D_{11}$ and $D_{12}$ are an N×6N matrix, n=1,2,…N. Their $n_{th}$ row matrices are

(30) $$h_{11}(n,1)=\left[\omega_{1}{\left(\theta_{n}\right)}^{T}s_{n}\right],h_{12}(n,:)=\left[{\omega_{2}}^{T}\left(\theta_{n},\beta_{n}\right)s_{n}\right],$$

(31) $$A_{11}(n,:)=\left[\omega_{1}{\left(\theta_{n}\right)}^{T},O_{1\times 6}\right],A_{12}(n,:)=\left[{\omega_{2}}^{T}\left(\theta_{n},\beta_{n}\right),O_{1\times 6}\right],$$

(32) $$D_{11}(n,:)=\left[O_{1\times 3(n-1)},\omega_{1}{\left(\theta_{n}\right)}^{T},O_{1\times (6N-3n)}\right],D_{12}(n,:)=\left[O_{1\times 3(n-1)},{\omega_{2}}^{T}\left(\theta_{n},\beta_{n}\right),O_{1\times (6N-3n)}\right],$$

where $h_{13}$ and $h_{14}$ are an (N−1)×1 matrix, $A_{13}$ and $A_{14}$ are an (N−1)×9 matrix, and $D_{13}$ and $D_{14}$ are ab (N−1)×6N matrix, n=2,3,…N. Their ${\left(n-1\right)}_{th}$ row matrices are

(33) $$h_{13}(n-1,:)=\left[s_{1}^{T}s_{1}-s_{n}^{T}s_{n}\right],h_{14}(n-1,:)=\left[s_{1}^{T}{\dot{s}}_{1}-s_{n}^{T}{\dot{s}}_{n}\right],$$

(34) $$\begin{array}{l} A_{13}(n-1,:)=-\left[2{\left(s_{n}-s_{1}\right)}^{T},O_{1\times 3},\tau_{n}^{2},2\tau_{n},0\right],\\ A_{14}(n-1,:)=-\left[{\left({\dot{s}}_{n}-{\dot{s}}_{1}\right)}^{T},{\left(s_{n}-s_{1}\right)}^{T},\tau_{n}{\dot{\tau }}_{n},{\dot{\tau }}_{n},\tau_{n}\right], \end{array}$$

(35) $$\begin{array}{ll} D_{13}(n-1,:)= & [-2\frac{R_{n}}{R_{1}}{\left(u^{o}-s_{1}\right)}^{T},O_{1\times 3(n-2)},2{\left(u^{o}-s_{n}\right)}^{T},O_{1\times (6N-3n)}],\\ D_{14}(n-1,:)= & \left[-{\left({\dot{u}}^{o}-{\dot{s}}_{1}+c^{o}\tau_{n}q_{u^{o},s_{1}}+c^{o}{\dot{\tau }}_{n}\rho_{u^{o},s_{1}}\right)}^{T},O_{1\times 3(n-2)},{\left({\dot{u}}^{o}-{\dot{s}}_{n}\right)}^{T},O_{1\times 3(N-n)},\right.\\ & \left.-\frac{R_{n}}{R_{1}}{\left(u^{o}-s_{1}\right)}^{T},O_{1\times 3(N-n)},{\left(u^{o}-s_{n}\right)}^{T},O_{1\times (3N-3n)}\right]. \end{array}$$

Similarly, in Equation (29), we have a (4N−2)×(4N−2) matrix of observation vector error coefficients, which can be represented in block matrix form as

(36) $$B_{1}=\mathrm{blkdiag}\left(B_{11},B_{12},B_{13}\right),$$

where

(37) $$\begin{array}{l} B_{11}=\mathrm{diag}([R_{1}\cos(\beta_{1}),R_{2}\cos(\beta_{2}),\ldots ,R_{N}\cos(\beta_{N})]),\\ B_{12}=\mathrm{diag}([R_{1},R_{2},\ldots ,R_{N}]),\\ B_{13}=\left[\begin{array}{cc} \mathrm{diag}(2[R_{2}c^{o},R_{3}c^{o},\ldots ,R_{N}c^{o}]) & O_{\left(N-1)\times (N-1\right)}\\ \mathrm{diag}([c^{o}{\dot{R}}_{2},c^{o}{\dot{R}}_{3},\ldots ,c^{o}{\dot{R}}_{N}]) & \mathrm{diag}([R_{2}c^{o},R_{3}c^{o},\ldots ,R_{N}c^{o}]) \end{array}\right]. \end{array}$$

5. Initial solution: Solving Equation (29) using weighted least squares (WLS) yields the estimated value $\psi_{1}$.

(38) $$\psi_{1}={\left(A_{1}^{T}W_{1}A_{1}\right)}^{-1}A_{1}^{T}W_{1}h_{1},$$

where the weighting matrix is $W_{1}={\left(B_{1}Q_{\gamma }B_{1}^{T}+D_{1}Q_{S}D_{1}^{T}\right)}^{-1}$.

According to (38), the calculation of the weighting matrix requires the use of an unknown parameter; we can replace $W_{1}$ with $Q_{\gamma }^{-1}$ to obtain the initial estimate and then update the weighted matrix using (32)–(37) to provide a more accurate estimate.

Then, we evaluate the covariance matrix of $\psi_{1}$, setting $\psi_{1}^{o}={\left(A_{1}^{T}W_{1}A_{1}\right)}^{-1}A_{1}^{T}W_{1}A_{1}\psi_{1}$, with $\Delta \psi_{1}$ as the estimation error of the first-step algorithm, to obtain

(39) $$\begin{array}{ll} \Delta \psi_{1} & =\psi_{1}-\psi_{1}^{o}={\left(A_{1}^{T}W_{1}A_{1}\right)}^{-1}A_{1}^{T}W_{1}(h_{1}-A_{1}\psi_{1}^{o})\\ & ={\left(A_{1}^{T}W_{1}A_{1}\right)}^{-1}A_{1}^{T}W_{1}(B_{1}\Delta \gamma +D_{1}\Delta S). \end{array}$$

If the sensor measurement error and platform system error are sufficiently small to be disregarded, the estimation of $\psi_{1}^{o}$ in the initial step of the proposed algorithm can be deemed unbiased [27]. Subsequently, the error covariance matrix of the estimation value obtained $\psi_{1}$ in the first step of the algorithm can be approximated as

(40) $$\mathrm{cov}(\psi_{1})=E(\Delta \psi_{1}\Delta \psi_{1})={\left(A_{1}^{T}W_{1}A_{1}\right)}^{-1}.$$

### 4.2. The Second-Step Calculation Principle

In the first step of the algorithm, due to neglecting the relationship between the introduced auxiliary variables and the target variable, information is lost. Therefore, in this step, we improve the accuracy of the target estimation obtained in the first-step solution by utilizing the correlation among the elements in the vector of unknown parameters $\psi_{1}^{o}$. Initially, we express the true values of the unknown parameters in terms of the initial estimates in the first step as follows

(41) $$u^{o}=\Psi_{1}(1:3)-\Delta \Psi_{1}(1:3),$$

(42) $${\dot{u}}^{o}=\Psi_{1}(4:6)-\Delta \Psi_{1}(4:6),$$

(43) $$c^{o2}=\Psi_{1}(7)-\Delta \Psi_{1}(7),$$

(44) $$R_{1}c^{o}=\Psi_{1}(8)-\Delta \Psi_{1}(8),$$

(45) $${\dot{R}}_{1}c^{o}=\Psi_{1}(9)-\Delta \Psi_{1}(9).$$

Moreover, there exists the following nonlinear relationship among the unknown vectors

(46) $$c^{o}\left\|u-s_{1}\right\|=R_{1}c^{o},$$

(47) $$\frac{c^{o}{\left(u^{o}-s_{1}\right)}^{T}\left({\dot{u}}^{o}-{\dot{s}}_{1}\right)}{R_{1}}={\dot{R}}_{1}c^{o}.$$

Substituting Equations (43) and (44) into Equation (46) and squaring both sides, while neglecting second- and higher-order terms, we obtain

(48) $$\left(u^{T}u+s_{1}^{T}s_{1}\right)\Psi_{1}(7)-\Psi_{1}^{2}(8)-2s_{1}^{T}\Psi_{1}(7)u=∥u-s_{1}{∥}^{2}\Delta \Psi_{1}(7)-2{\hat{\Psi }}_{1}(8)\Delta \Psi_{1}(8).$$

Expanding $u^{T}u$ and $∥u-s_{1}{∥}^{2}$ around $\Psi_{1}(1:3)$ using Taylor series yields

(49) $$\begin{array}{c} u^{T}u\approx \Psi_{1}^{T}(1:3)\Psi_{1}(1:3)-2\Psi_{1}^{T}(1:3)\Delta \Psi_{1}(1:3),\\ ∥u-s_{1}{∥}^{2}\approx ∥\Psi_{1}(1:3)-s_{1}{∥}^{2}-2{\left(\Psi_{1}(1:3)-s_{1}\right)}^{T}\Delta \Psi_{1}(1:3). \end{array}$$

Substituting (49) into (48) yields

(50) $$\begin{array}{l} \left(\Psi_{1}^{T}(1:3)\Psi_{1}(1:3)+s_{1}^{T}s_{1}\right)\Psi_{1}(7)-\Psi_{1}^{2}(8)-2s_{1}^{T}\Psi_{1}(7)u\\ =2\Psi_{1}(7)\Psi_{1}^{T}(1:3)\Delta \Psi_{1}(1:3)+∥\Psi_{1}(1:3)-s_{1}{∥}^{2}\Delta \Psi_{1}(7)-2\Psi_{1}(8)\Delta \Psi_{1}(8). \end{array}$$

Similarly, we multiply both sides of (47) by $c^{o}$ at the same time and substitute (43)–(45) into (47) to obtain:

(51) $$\Psi_{1}(7)\left(u^{oT}{\dot{u}}^{o}-{\dot{s}}_{1}^{T}u-s_{1}^{T}{\dot{u}}^{o}+s_{1}^{T}{\dot{s}}_{1}\right)={\left(u^{o}-s_{1}\right)}^{T}\left({\dot{u}}^{o}-{\dot{s}}_{1}\right)\Delta \Psi_{1}(7)-\Psi_{1}(9)\Delta \Psi_{1}(8)-\Psi_{1}(8)\Delta \Psi_{1}(9).$$

Expanding $u^{oT}{\dot{u}}^{o}$ and ${\left(u^{o}-s_{1}\right)}^{T}\left({\dot{u}}^{o}-{\dot{s}}_{1}\right)$ around $\Psi_{1}(1:3)$ and $\Psi_{1}(4:6)$ using Taylor series yields

(52) $$\begin{array}{c} u^{oT}{\dot{u}}^{o}\approx \Psi_{1}^{T}(1:3)\Psi_{1}(4:6)-\Psi_{1}^{T}(4:6)\Delta \Psi_{1}(1:3)-\Psi_{1}^{T}(1:3)\Delta \Psi_{1}(4:6),\\ \begin{array}{l} {\left(u^{o}-s_{1}\right)}^{T}\left({\dot{u}}^{o}-{\dot{s}}_{1}\right)\approx {\left(\Psi_{1}(1:3)-s_{1}\right)}^{T}\left(\Psi_{1}(4:6)-{\dot{s}}_{1}\right)-\\ {\left({\dot{u}}^{o}-{\dot{s}}_{1}\right)}^{T}\Delta \Psi_{1}(1:3)-{\left(u^{o}-s_{1}\right)}^{T}\Delta \Psi_{1}(4:6). \end{array} \end{array}$$

Substituting (52) into (51) yields

(53) $$\begin{array}{l} \Psi_{1}(7)(\Psi_{1}^{T}(1:3)\Psi_{1}(4:6)+s_{1}^{T}{\dot{s}}_{1})-\Psi_{1}(9)\Psi_{1}(8)-\Psi_{1}(7)({\dot{s}}_{1}^{T}u^{o}+s_{1}^{T}{\dot{u}}^{o})=\Psi_{1}(7)\Psi_{1}^{T}(4:6)\Delta \Psi_{1}(1:3)+\\ \Psi_{1}(7)\Psi_{1}^{T}(1:3)\Delta \Psi_{1}(4:6)+{\left(\Psi_{1}(1:3)-s_{1}\right)}^{T}\left(\Psi_{1}(4:6)-{\dot{s}}_{1}\right)\Delta \Psi_{1}(7)-\Psi_{1}(9)\Delta \Psi_{1}(8)-\Psi_{1}(8)\Delta \Psi_{1}(9). \end{array}$$

Combining (41)–(43) and (50) and (53), the matrix expression for the second step of the algorithm can be obtained

(54) $$h_{2}-A_{2}\psi_{2}^{o}=B_{2}\Delta \Psi_{1}.$$

In (54), the unknown vector parameter is $\psi_{2}^{o}={\left[u^{oT},{\dot{u}}^{oT},c^{o2}\right]}^{T}$, $h_{2}$ is the 9×1 measurement vector, $A_{2}$ is the 9×7 measurement matrix, and $B_{2}$ is the 9×9 error coefficient matrix, which is defined as

(55) $$\begin{array}{l} h_{2}=\left[{\hat{\Psi }}_{1}^{T}(1:7),\left({\hat{\Psi }}_{1}^{T}(1:3){\hat{\Psi }}_{1}(1:3)+s_{1}^{T}s_{1}\right){\hat{\Psi }}_{1}(7)-{\hat{\Psi }}_{1}^{2}(8),\right.\\ \left.\Psi_{1}(7)\left(\Psi_{1}^{T}(1:3)\Psi_{1}(4:6)+s_{1}^{T}{\dot{s}}_{1}\right)-\Psi_{1}(9)\Psi_{1}(8)\right], \end{array}$$

(56) $$A_{2}=\left[\begin{array}{ccc} I_{3\times 3} & O_{3\times 3} & O_{3\times 1}\\ O_{3\times 3} & I_{3\times 3} & O_{3\times 1}\\ O_{1\times 3} & O_{1\times 3} & 1\\ 2s_{1}^{T}{\hat{\Psi }}_{1}(7) & O_{1\times 3} & 0\\ {\dot{s}}_{1}^{T}{\hat{\Psi }}_{1}(7) & s_{1}^{T}{\hat{\Psi }}_{1}(7)\; & 0 \end{array}\right],$$

(57) $$B_{2}=\left[\begin{array}{lllll} & I_{7\times 7} & & O_{7\times 2} & \\ 2{\hat{\Psi }}_{1}(7){\hat{\Psi }}_{1}^{T}(1:3) & O_{1\times 3} & ∥{\hat{\Psi }}_{1}(1:3)-s_{1}{∥}^{2} & -2{\hat{\Psi }}_{1}(8) & 0\\ \Psi_{1}(7)\Psi_{1}^{T}(4:6) & {\hat{\Psi }}_{1}(7){\hat{\Psi }}_{1}^{T}(1:3) & {\left(\Psi_{1}(1:3)-s_{1}\right)}^{T}\left(\Psi_{1}(4:6)-{\dot{s}}_{1}\right) & -\Psi_{1}(9) & -{\hat{\Psi }}_{1}(8) \end{array}\right].$$

Solving (50) using the WLS algorithm yields the estimate $\psi_{2}$ of $\psi_{2}^{o}$ as follows

(58) $$\psi_{2}={\left(A_{2}^{T}W_{2}A_{2}\right)}^{-1}A_{2}^{T}W_{2}h_{2},$$

where the weighting matrix is $W_{2}={\left(B_{2}\mathrm{cov}(\psi_{1})B_{2}^{T}\right)}^{-1}$.

Let $\psi_{2}^{o}={\left(A_{2}^{T}W_{2}A_{2}\right)}^{-1}A_{2}^{T}W_{2}A_{2}\psi_{2}^{o}$; subtracting $\psi_{2}^{o}$ from $\psi_{2}$, we obtain the estimation error $\Delta \psi_{2}$ of the second-step algorithm

(59) $$\Delta \psi_{2}={\left(A_{2}^{T}W_{2}A_{2}\right)}^{-1}A_{2}^{T}W_{2}B_{2}\Delta \Psi_{1}.$$

From (59), it is evident that $\Delta \psi_{2}$ and $\Delta \psi_{1}$ are linearly correlated, implying unbiased estimation of the unknown variable $\psi_{2}^{o}$. Thus, the covariance matrix of the estimate $\psi_{2}$ obtained using the second-step algorithm can be approximated as follows

(60) $$\mathrm{cov}(\psi_{2})=E(\Delta \psi_{2}\Delta \psi_{2}^{T})={\left(A_{2}^{T}W_{2}A_{2}\right)}^{-1}.$$

Finally, the valuation of the position and velocity of the target source as well as the propagation speed of the signal are

(61) $$\left\{\begin{array}{c} u=\psi_{2}(1:3)\\ \dot{u}=\psi_{2}(4:6)\\ c=\sqrt{\psi_{2}(7)} \end{array}\right..$$

### 4.3. Algorithmic Steps

We summarize the proposed algorithm in Algorithm 1.
**Algorithm 1:** The underwater moving target localization algorithm proposed in this paper**Input:** Information on the position and speed parameters of the moving platforms S, the observation vectors $\gamma ={\left[\theta^{T},\beta^{T},\tau^{T},{\dot{\tau }}^{T}\right]}^{T}$, the measurement error covariance matrix $Q_{\gamma }$, the platform systematic error covariance matrix $Q_{s}$. **First-step algorithm:**Compute the observation vector matrix $h_{1}$ and the pseudo-linear observation vector coefficient matrix $A_{1}$ using (32)–(35).Initialize $W_{1}=Q_{\gamma }^{-1}$, and compute an initial estimate of $\Psi_{1}$ using (38).Calculate $B_{1}$ and $D_{1}$ using an initial estimate of $\Psi_{1}$ and (36)–(37).Recalculate the weighting matrix $W_{1}$.Calculate the new estimate of $\Psi_{1}$ using (38). **Second-step algorithm:**6.Calculate the error covariance matrix $\mathrm{cov}(\psi_{1})$ using (40).7.Calculate $B_{2}$ using $\psi_{1}$ and (57); then, obtain the weighting matrix $W_{2}$.8.Calculate $h_{2}$ using (55).9.Calculate the estimation vector $\psi_{2}$ using (58).10.Calculate $u,\dot{u},c$ using (61).**Output:** Target position and velocity and acoustic source signal velocity.

## 5. Algorithm Analysis

### 5.1. Algorithm Performance Analysis

This summary will testify to the theoretical performance of the proposed algorithm in realizing the CRLB. We will derive the covariance matrix of the unknown vector estimates $\psi_{2}$ using the proposed algorithm and compare and analyze it with (15) to determine whether it is equal. Firstly, the algorithm in this paper is applied under the conditions that the position error, velocity error, and sensor measurement error of the mobile platform are small, i.e., small errors, and therefore satisfy

(62) $$\begin{array}{l} C1:\left\|\Delta \theta_{n}\right\|\ll \left\|\theta_{n}^{o}\right\|,\left\|\Delta \beta_{n}\right\|\ll \left\|\beta_{n}^{o}\right\|,\\ C2:\left\|\Delta \tau_{n}\right\|\ll \left\|\tau_{n}^{o}\right\|,\\ C3:\left\|\Delta {\dot{\tau }}_{n}\right\|\ll \left\|{\dot{\tau }}_{n}^{o}\right\|,\\ C4:\left\|\Delta s_{n}\right\|\ll \left\|s_{n}^{o}\right\|,\left\|\Delta {\dot{s}}_{n}\right\|\ll \left\|{\dot{s}}_{n}^{o}\right\|. \end{array}$$

Conditions C1 and C4 are achievable with small bearing and platform systematic errors; C2 can be met with small time delay differences or distant targets; and C3 is attainable with small frequency difference errors or slow-moving targets. In underwater environments, object speeds typically correspond to a few meters per second, thus making Conditions C2 and C3 suitable for underwater localization within a few kilometers. Furthermore, these error conditions are commonly employed in the existing literature [31,32,33,35,36].

We define the estimate of the unknown parameter as ν and the estimate of the target unknown parameter as φ. Then, the unknown vector estimation error is $\Delta \nu ={\left[\Delta \varphi^{T},\Delta c\right]}^{T}$, where $\Delta \varphi =\varphi -\varphi^{o}$, $\Delta c=c-c^{o}$, which is carried into (61), and Δφ can be expressed according to the estimation error $\Delta \psi_{2}$ in the second step of the proposed algorithm as

(63) $$\Delta \varphi =\psi_{2}(1:6)-\psi_{2}^{o}(1:6)=\Delta \psi_{2}(1:6).$$

Expanding $\psi_{2}^{o}(7)$ around c using Taylor series yields

(64) $$\Delta \psi_{2}(7)=\psi_{2}(7)-\psi_{2}^{o}(7)=2c\Delta c.$$

Therefore, the unknown variable error Δν can be related to $\Delta \psi_{2}$ as follows

(65) $$\Delta \nu =B_{3}\Delta \psi_{2},$$

where $B_{3}=\mathrm{diag}\left(1,1,1,1,1,1,\frac{1}{2c}\right)$.

Under small error conditions, Δν is linearly correlated with $\Delta \psi_{2}$ from (65), and the estimate of $\psi_{2}^{o}$ is unbiased, so the final estimate of the unknown parameter ν is unbiased. The covariance matrix of ν can be approximated as

(66) $$\mathrm{cov}(\nu )=E(\Delta \nu \Delta \nu^{T})=B_{3}\mathrm{cov}(\psi_{2})B_{3}^{T}=B_{3}{\left(B_{2}\mathrm{cov}(\psi_{1})B_{2}^{T}\right)}^{-1}B_{3}^{T}.$$

By substituting $W_{1}={\left(B_{1}Q_{\gamma }B_{1}^{T}+D_{1}Q_{S}D_{1}^{T}\right)}^{-1}$ into (62) and utilizing the matrix inversion lemma [37], we obtain

(67) $$\mathrm{cov}(\nu )={\left(B_{3}^{-T}A_{2}^{T}B_{2}^{-T}A_{1}^{T}W_{1}A_{1}B_{2}^{-1}A_{2}B_{3}^{-1}\right)}^{-1}.$$

According to the Sherman–Morrison–Woodbury formula, expanding $W_{1}$ yields

(68) $$W_{1}=B_{1}^{-T}Q_{\gamma }^{-1}B_{1}^{-1}-B_{1}^{-T}Q_{\gamma }^{-1}B_{1}^{-1}D_{1}{\left(Q_{S}^{-1}+D_{1}^{T}B_{1}^{-T}Q_{\gamma }^{-1}B_{1}^{-1}D_{1}\right)}^{-1}D_{1}^{T}B_{1}^{-T}Q_{\gamma }^{-1}B_{1}^{-1}.$$

Substituting (68) into (67) to obtain

(69) $$\mathrm{cov}(\nu )={\left(G_{1}^{T}Q_{\gamma }^{-1}G_{1}-G_{1}^{T}Q_{\gamma }^{-1}G_{2}{\left(Q_{S}^{-1}+G_{2}^{T}Q_{\gamma }^{-1}G_{2}\right)}^{-1}G_{2}^{T}Q_{\gamma }^{-1}G_{1}\right)}^{-1},$$

where $G_{1}=B_{1}^{-1}A_{1}B_{2}^{-1}A_{2}B_{3}^{-1}$,$G_{2}=B_{1}^{-1}D_{1}$.

Comparing (69) and (15), it is seen that they have the same form, so it is necessary to compare the relationship between $G_{1}$ and $\left[\frac{\partial \gamma^{o}}{\partial \varphi^{o}}\;\frac{\partial \gamma^{o}}{\partial c^{o}}\right]$ and $G_{2}$ and $\frac{\partial \gamma^{o}}{\partial S^{o}}$, which can be obtained using (58). We can obtain

(70) $$G_{1}\approx \left[\frac{\partial \gamma^{o}}{\partial \varphi^{o}}\;\frac{\partial \gamma^{o}}{\partial c^{o}}\right],G_{2}\approx -\frac{\partial \gamma^{o}}{\partial S^{o}}.$$

According to (66), it can be shown that $\mathrm{cov}(\nu )\approx \mathrm{CLRB}\left(\nu^{o}\right)$, and the target position, velocity, and speed of sound estimated using the proposed algorithm can reach the CRLB under this localization model. The detailed calculation procedure for (66) is given in Appendix B.

### 5.2. Algorithmic Complexity Analysis

This subsection provides a theoretical analysis of the computational complexity of the proposed algorithm, focusing on the required number of real multiplications. Assuming m×p matrices are multiplied by p×q matrices, the computational effort is denoted as mpq. The computation of n×n matrix inversion is denoted by $n^{3}$. The computational complexity of the first step of the algorithm proposed in this paper mainly consists of calculating the weighting matrix $W_{1}$ and the initial solution $\psi_{1}$. Calculating $W_{1}$ requires the computation of several arithmetic units, which are $B_{1}Q_{\gamma }$, $B_{1}Q_{\gamma }B_{1}^{T}$, $D_{1}Q_{S}$, $D_{1}Q_{S}D_{1}^{T}$, and ${\left(B_{1}Q_{\gamma }B_{1}^{T}+D_{1}Q_{S}D_{1}^{T}\right)}^{-1}$; solving for $\psi_{1}$ consists of $A_{1}^{T}W_{1}$, $A_{1}^{T}W_{1}A_{1}$,${\left(A_{1}^{T}W_{1}A_{1}\right)}^{-1}$, ${\left(A_{1}^{T}W_{1}A_{1}\right)}^{-1}A_{1}^{T}W_{1}$, and ${\left(A_{1}^{T}W_{1}A_{1}\right)}^{-1}A_{1}^{T}W_{1}h_{1}$. The computational complexity of the second step mainly focuses on the computation of $W_{2}$, which includes $B_{2}\mathrm{cov}(\psi_{1})$, $B_{2}\mathrm{cov}(\psi_{1})B_{2}^{T}$, ${\left(B_{2}\mathrm{cov}(\psi_{1})B_{2}^{T}\right)}^{-1}$, and $\psi_{2}$, which include $A_{2}^{T}W_{2}$, $A_{2}^{T}W_{2}A_{2}$, ${\left(A_{2}^{T}W_{2}A_{2}\right)}^{-1}$, ${\left(A_{2}^{T}W_{2}A_{2}\right)}^{-1}A_{2}^{T}W_{2}$, and ${\left(A_{2}^{T}W_{2}A_{2}\right)}^{-1}A_{2}^{T}W_{2}h_{2}$.

The complexity of each unit of the proposed algorithm combining the AOA/TDOA/FDOA is given in Table 2, and all the (4N−2) matrices in the table need to be replaced by (2N−2) matrices if only the computational complexity of the TDOA/FDOA observations is utilized.

Compared to references [22,32,36], our algorithm is optimized for computational complexity. Compared to [36], both algorithms have the same computational complexity for $W_{1}$ and $\psi_{1}$, both being ${\left(2N-2\right)}^{2}(12N+3)$ and $\left(2N-2)(36N^{2}+171\right)$. However, [36] requires iterations in solving $W_{2}$ and introduces more unknown variables, resulting in increased computational complexity in the second step compared to our algorithm. Ref [32] proposes a four-step solution using only TDOA observations, allowing estimation of the target position, but the four-step calculation process increases the overall computational load. The SDP solution proposed in [22] introduces N additional unknowns and requires multiple iterations, making it the most computationally demanding. In summary, the computational complexity of the second-step correction in the proposed algorithm is lower than that of existing closed-form algorithms. This is because the additional variable count is reduced to 2 in the first step of our algorithm, the second step only considers the relationship between additional variables and unknowns, and the matrix dimension is independent of the number of platforms, thereby reducing the computational complexity.

## 6. Simulation Experiments

In this section, the theoretical analysis of the proposed algorithm is further validated through Monte Carlo (MC) simulation experiments. We compare the proposed algorithm with three classical methods: the SDP method [22], four-step WLS methods (FS-WLS) [32], and two-step WLS methods (TS-WLS) [36]. Although the localization scenarios for some algorithms differ from those in this paper, these three localization algorithms cover a wide range of scenarios, including the speed of sound being known or unknown and taking systematic errors into account or not, and possess a high degree of novelty themselves. Hence, they are highly comparable to the algorithms in this paper according to several key dimensions, facilitating a comprehensive and in-depth evaluation of the algorithms’ performance. The propagation speed of sound in seawater generally ranges between 1420 m/s and 1560 m/s [29]. Hence, the unknown speed of sound in each of the following simulation scenarios is randomly generated within the range of (1420,1560) m/s with a uniform distribution. Additionally, all the simulation experiments are evaluated using the root mean square error (RMSE) in each simulation, calculated as follows:

(71) $$\mathrm{RMSE}(\nu )=\sqrt{\sum_{n=1}^{M}{\left\|\nu^{n}-\nu^{o}\right\|}^{2}/M},$$

where $\nu^{n}$ is the estimate of $\nu^{o}$ from the $n_{\mathrm{th}}$ simulation experiment, and the total number of MC simulation experiments is M=5000. All the RMSE results are shown in dB; in addition, the square root CRLBs as the performance bounds are also given in the simulation. In addition, the simulation experiments are carried out using MATLAB2022 software.

### 6.1. Effect of Measurement Errors on Positioning Performance

**Figure 6:**
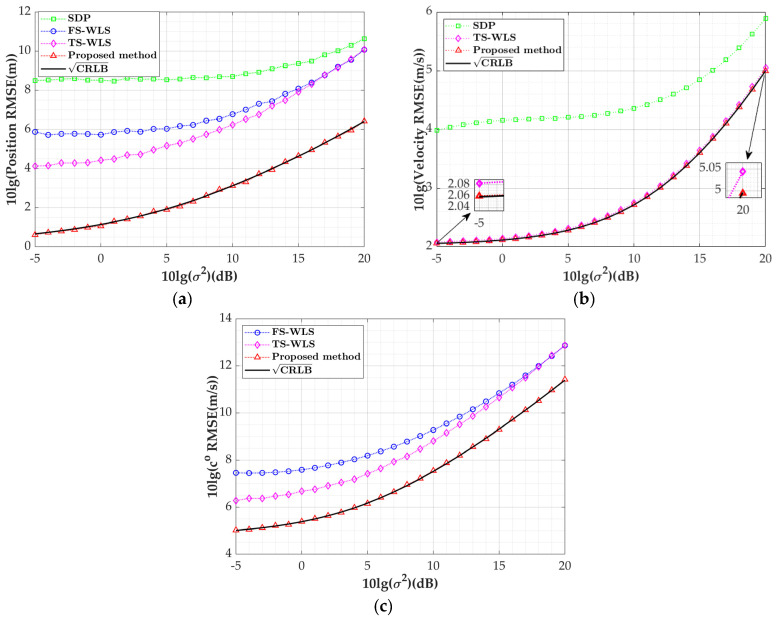
Variation curve of target parameter estimation RMSEs with sensor measurement errors: (**a**) position estimation RMSE; (**b**) velocity estimation RMSE; (**c**) speed of sound estimation RMSE.

This subsection will investigate the performance of the proposed algorithm as the sensor measurement error varies, consistent with the target’s position in Section 2. To emphasize the variation in the velocity, the speeds of high-speed underwater targets such as submarines and torpedoes are adopted as ${\dot{u}}^{o}={\left[-20,-15,-20\right]}^{T}m/s$ [31], with the systematic error set to $\sigma_{s}=1\;m$, and the observation disturbance parameter varies from −5 dB to 20 dB. Since the FS-WLS algorithm only uses TDOA observations, it estimates only the target position and sound speed. In the SDP method, the speed of sound is known. In the simulation, we take $c=c^{o}+20$ as the known sound speed and use the MATLAB toolbox CVX to solve the SDP problems [38]. Figure 6a–c, respectively, depict the RMSE curves of the position estimation, velocity estimation, and speed of sound estimation as the sensor measurement error varies.

Based on the simulation results, the positioning algorithm proposed in this paper can achieve estimates of the target position, velocity, and speed of sound within the entire range of $\sigma^{2}$ with corresponding accuracy in the CRLB, outperforming the other three algorithms. The SDP algorithm performs lower in its target position and velocity estimations compared to the other algorithms, indicating the significance of estimating the speed of sound to enhance the algorithm performance. From Figure 6a,b, it is evident that the FS-WLS algorithm exhibits a lower estimation RMSE under small error conditions because it only utilizes the TDOA for positioning and does not consider system errors. In Figure 6b, the velocity estimation RMSE of this algorithm is slightly higher than that of the FS-WLS algorithm due to the target velocity estimation being primarily influenced by the FDOA equation, with the target velocity being significantly smaller than the position value, resulting in a minimal difference in the velocity estimation between the two algorithms. Since the FS-WLS algorithm can estimate three parameters, the data from Figure 6a–c are extracted into Table 3 for comparison with this algorithm. At an SNR of −5 dB, the TS-WLS algorithm differs from this algorithm by 3.46 dB in its position estimation RMSE, 0.02 dB in its sound speed estimation RMSE, and 1.17 dB in its velocity estimation RMSE; meanwhile, at an SNR of 20 dB, the difference in the position estimation RMSE between the two algorithms increases to 3.60 dB, the velocity estimation RMSE difference rises to 0.06 dB, and the sound speed estimation RMSE difference reaches 1.48 dB. As the measurement errors increase, the differences in the estimations between the two algorithms gradually grow, indicating that this algorithm demonstrates stronger robustness in response to varying measurement errors.

### 6.2. Algorithm for the Effect of Systematic Errors on Positioning Performance

**Figure 7:**
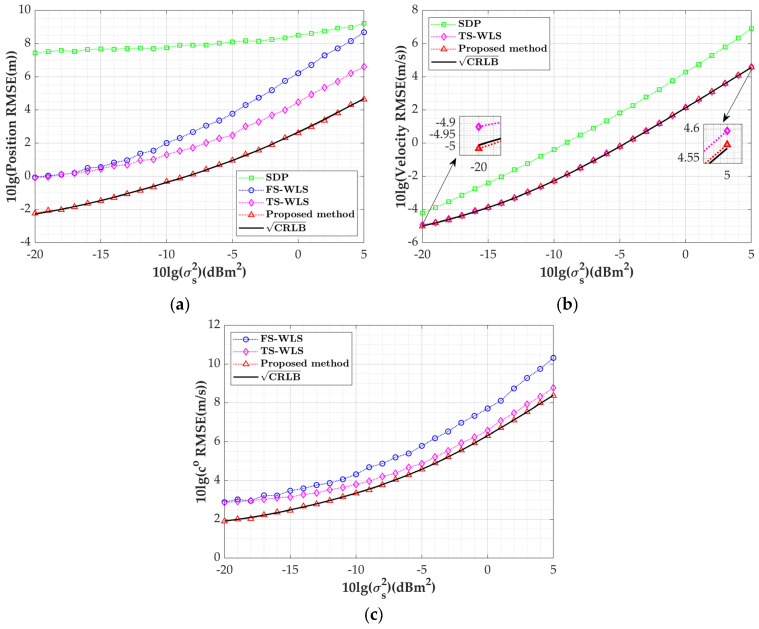
Variation curve of target parameter estimation RMSE with systematic error: (**a**) position estimation RMSE; (**b**) velocity estimation RMSE; (**c**) sound speed estimation RMSE.

In this subsection, we will consider the variation in the proposed algorithm’s performance with the systematic error of the mobile platform by setting the sensor observation perturbation parameter $\sigma_{\mathrm{aoa}}=1\;\deg$ and $\sigma_{d}=1\;m$. The systematic error is varied from −5 dB to 20 dB, and the rest of the simulation conditions remain unchanged; the simulation results are shown in Figure 7a–c.

Similarly, the estimated RMSE of the proposed algorithm in this paper achieves the CRLB with variations in the systematic error and outperforms the other three algorithms. From Figure 7a, it is evident that when $\sigma_{s}^{2}\leq 0\;\mathrm{dB}$, the position estimation RMSE of the algorithm proposed in this paper is at least 6 dB lower than that of the SDP algorithm, which does not consider errors in the speed of sound, and at least 2 dB lower than that of the TS-WLS algorithm. Comparing Figure 7b,c, it can be seen that as the systematic error increases, the trends in the growth of the speed estimation RMSE of the SDP algorithm and the sound speed estimation RMSE of the FS-WLS algorithm are more significant than those of the other two algorithms. This is mainly due to the fact that the SDP algorithm ignores the effect of sound velocity conditions in its operation, while the FS-WLS algorithm does not fully consider systematic errors. It is also noted that the performance gap between the TS-WLS algorithm and the algorithm in this paper decreases because both algorithms ignore second-order errors. When the error increases to a certain point, this approximation method will no longer be accurate. Nevertheless, under small error conditions, the algorithm in this paper still outperforms the TS-WLS algorithm.

### 6.3. Error Cumulative Distribution Function (CDF) Comparison

The interaction between the mobile platform and the shape of the target typically affects the positioning performance. We will analyze in detail the performance of the proposed algorithm compared to existing algorithms using different position distributions of the target and the mobile platform by evaluating the cumulative distribution function (CDF) of the RMSE. We assume 10 mobile platforms and 1 target source are randomly distributed in an $([-1000,1000]\times [-1000,1000]\times [-1000,1000])\;m^{2}$ underwater cubic space, with the speed parameters of the mobile platforms and the target randomly generated within the range of [-5,5] m/s to simulate uncertainty in real-world environments. The experiment randomly generates 1000 scenarios, with the RMSE of the target parameter estimates in each scenario used as the simulation data. The sensor measurement error is set to $\sigma_{\mathrm{aoa}}=1\;\deg$, $\sigma_{d}=1\;m$, the system error is set to $\sigma_{s}=1\;m$, and the MC simulation is carried out 5000 times for each scenario in the simulation.

**Figure 8:**
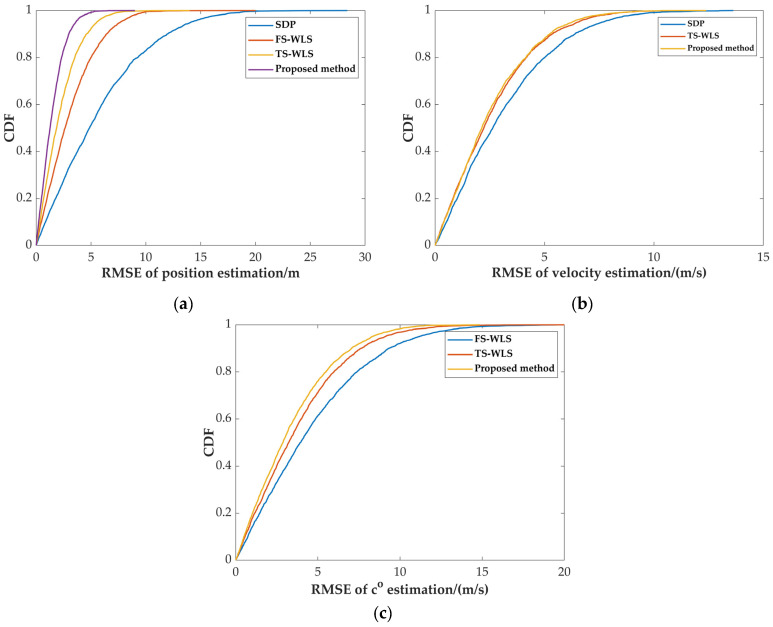
Comparison of CDF plots for different algorithms: (**a**) position estimation; (**b**) velocity estimation; (**c**) sound speed estimation.

Figure 8 depicts the cumulative distribution function (CDF) plots of the RMSEs for the target source position, velocity, and sound speed estimation. Observing Figure 8a, it is evident that the abscissa corresponding to the curve of the proposed algorithm is significantly smaller than that of the other three algorithms, even at the same CDF value. This indicates that in the simulation environment in this section, the RMSE in the target position estimation of the proposed algorithm is significantly smaller than that of the other three algorithms, even at the same probability level. Similarly, the conclusions drawn from Figure 8b,c indicate that the proposed algorithm performs comparably to the TS-WLS algorithm in velocity estimation but outperforms the SDP algorithm. Additionally, in sound speed estimation, the RMSE of the proposed algorithm is superior to that of the TS-WLS and FS-WLS algorithms.

### 6.4. Effect of the Number of Mobile Platforms on Localization Performance

The number of mobile platforms also affects the target localization performance. Therefore, this section evaluates the performance of the proposed algorithm under different numbers of mobile platforms. The simulation conditions are the same as those set in Section 6.3, with a randomly selected localization scenario. The number of mobile platforms is set to N=4,5,…10.

**Figure 9:**
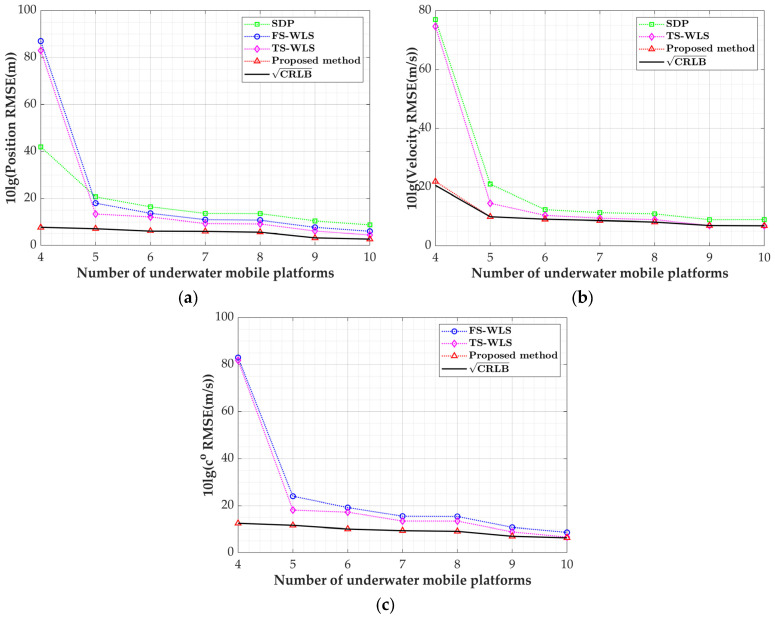
Variation in target parameter estimation RMSE with the number of moving platforms: (**a**) position estimation RMSE; (**b**) velocity estimation RMSE; (**c**) sound speed estimation RMSE.

Figure 9 depicts how varying the number of mobile platforms affects the localization performance in random scenarios. The simulation results show that the proposed algorithm closely approximates the CRLB and outperforms the other three algorithms. As illustrated in Figure 9a, the performance of the proposed algorithm is comparable to that of other algorithms with eight platforms when there are only four mobile platforms. With fewer mobile platforms, the performance gap between the proposed algorithm and the other algorithms widens. This is because the other algorithms introduce more auxiliary variables and have fewer measurements, which makes it impossible to obtain a unique solution with fewer observation platforms, leading to a greater performance loss. The algorithm proposed in this study utilizes AOA observations and only needs to meet a certain condition to achieve target localization.

### 6.5. Effect of Target Position on Localization Performance

**Figure 10:**
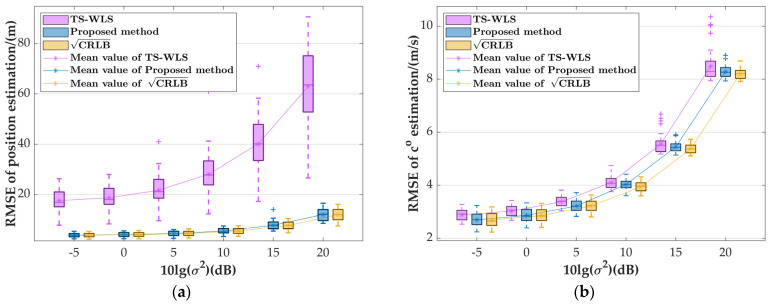
Boxplot of the variation in the parameter estimation RMSE of random far-field target sources with measurement errors: (**a**) position estimation; (**b**) speed of sound estimation.

The localization performance is significantly affected by the target’s location, especially its distance from the moving platform. Since the experiments focused on near-field targets, this subsection will investigate the impact of far-field targets on the proposed algorithm’s performance. Assuming the localization scenario described below, where the moving platform’s position and velocity parameters match those in Table 1, we define a far-field cubic region with dimensions within a range of [2000,3000] m along the x, y, and z axes and randomly place 50 target sources within this region. The systematic error is set to $\sigma_{s}=1\;m$, while $\sigma^{2}$ is adjusted from −5 dB to 20 dB. Additionally, 5000 MC simulations are conducted at each location. Figure 10a,b present boxplots illustrating the estimated RMSEs of the target source at 50 random positions and the estimated RMSEs of the corresponding speed of sound considering measurement errors, respectively.

Figure 10 illustrates that the box maximum and minimum values for the estimated RMSEs and CRLBs from our algorithm are remarkably close. For instance, when $\sigma^{2}=20\;\mathrm{dB}$, the maximum RMSE for position estimation and its corresponding CRLB are 16.4537 m and 16.0758 m, respectively, with a difference of merely 0.3779 m. Similarly, the minimum values are 8.5482 m and 7.5654 m, differing by 0.9828 m, while the average values are 12.0175 m and 12.0354 m, differing by only 0.0179 m. In contrast, for the TS-WLS algorithm, the maximum, minimum, and mean values are significantly higher, at 90.643 m, 26.5601 m, and 62.9327 m, respectively, compared to 74.18 m, 18.0119 m, and 50.9152 m for our algorithm. Furthermore, Figure 10b reveals that our algorithm’s sound speed estimation RMSE deviates by 0.0863 m/s and 1.5868 m/s from the maximum values of the corresponding CRLB and TS-WLS algorithms, respectively. It also deviates by 0.0230 m/s and 0.0206 m/s from the minimum values and by 0.0719 m/s and 0.3002 m/s from the mean values. The analysis above clearly demonstrates that within a specific range of measurement errors, for various far-field target positions, our algorithm achieves estimated RMSE values for the target position and speed of sound that closely match the corresponding CRLB and outperform the TS-WLS algorithm.

The sensor’s error is set to $\sigma_{\mathrm{aoa}}=1\;\deg$, $\sigma_{d}=1\;m$, the systematic error is varied from −20 dB to 5 dB, and the other simulation parameters are unchanged.

**Figure 11:**
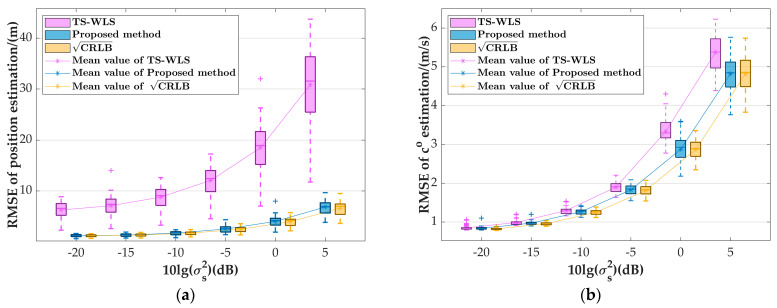
Boxplot of the variation in the parameter estimation RMSE of random far-field target source with systematic errors: (**a**) position estimation; (**b**) speed of sound estimation.

Figure 11 depicts a boxplot of the target source parameter estimation RMSE as a function of the systematic error variation. It can be observed from Figure 11 that within the entire $\sigma_{s}^{2}$ range of variation, the maximum, minimum, and mean values of the boxplot of our algorithm are close to the corresponding CRLB, and all are lower than those of the TS-WLS algorithm. Therefore, we can conclude that within a reasonable range of systematic errors, the proposed algorithm demonstrates asymptotic optimality in its position and sound speed estimation for far-field target sources at different positions, and it significantly outperforms the TS-WLS algorithm.

## 7. Conclusions

In this paper, a new two-step closed-form localization algorithm with a joint AOA/TDOA/FDOA method is proposed to estimate the position and velocity of a target sound source and its signal propagation velocity given its uncertainty in an underwater environment, as well as the position and velocity errors for moving platforms. The algorithm first introduces auxiliary variables in the first stage, obtaining initial solutions by constructing pseudo-linear equations; then, in the second stage, based on the relationship between the auxiliary variables and unknown variables, it further obtains exact solutions containing only unknown variables. Through in-depth theoretical analysis and simulation experiments, the proposed algorithm achieves a localization accuracy comparable to the precision of the CRLB when the sensor measurement errors and mobile platform system errors are within reasonable ranges. The experiments also demonstrate that even in scenarios with fewer mobile platforms, uncertain platform–target geometries, and far-field sound source targets, the algorithm can still achieve effective localization. Moreover, the root mean square errors of the estimated target sound source position, velocity, and sound propagation speed are superior to those of existing algorithms, demonstrating its estimation accuracy.

This study considers the target sound source as the point source. In reality, the physical properties of the sounding object, like its shape and size, often influence the sound wave propagation, impacting the localization accuracy. Future research will delve into factors such as the shape and size of the target object, examining their effects on the localization performance and refining the localization algorithm accordingly.

## Figures and Tables

**Table 1:** Real position and velocity parameters of moving platforms.

**Platform** no.n	1	2	3	4	5	6	7	8	9	10
$x_{n}^{o}$	0	0	0	0	1000	1000	1000	1000	500	500
$y_{n}^{o}$	100	0	0	1000	1000	1000	1000	1000	500	500
$z_{n}^{o}$	0	0	1000	1000	0	0	1000	1000	1000	0
${\dot{x}}_{n}^{o}$	3	−3	1	1	2	2	1.2	−1.5	1.3	2.5
${\dot{y}}_{n}^{o}$	−2	1	−2	2	−1	−1	−1.5	1.2	1.3	2.5
${\dot{z}}_{n}^{o}$	2	−2	1	3	1	1	1.5	−1.2	1.3	2.5

**Table 2:** Complexity of each unit of the proposed algorithm.

Calculation Unit		Unit Complexity	Total Unit Complexity
$W_{1}$	$B_{1}Q_{\gamma }$	${\left(4N-2\right)}^{3}$	$\begin{array}{l} 3{\left(4N-2\right)}^{3}+\\ 36(4N-2)N^{2}+\\ 6{\left(4N-2\right)}^{2}N \end{array}$
	$B_{1}Q_{\gamma }B_{1}^{T}$	${\left(4N-2\right)}^{3}$	
	$D_{1}Q_{S}$	$36(4N-2)N^{2}$	
	$D_{1}Q_{S}D_{1}^{T}$	$6{\left(4N-2\right)}^{2}N$	
	${\left(B_{1}Q_{\gamma }B_{1}^{T}+D_{1}Q_{S}D_{1}^{T}\right)}^{-1}$	${\left(4N-2\right)}^{3}$	
$\psi_{1}$	$A_{1}^{T}W_{1}$	$9{\left(4N-2\right)}^{2}$	$\begin{array}{l} 9{\left(4N-2\right)}^{2}+\\ 171(4N-2)+\\ 729 \end{array}$
	$A_{1}^{T}W_{1}A_{1}$	81(4N−2)	
	${\left(A_{1}^{T}W_{1}A_{1}\right)}^{-1}$	729	
	${\left(A_{1}^{T}W_{1}A_{1}\right)}^{-1}A_{1}^{T}W_{1}$	81(4N−2)	
	${\left(A_{1}^{T}W_{1}A_{1}\right)}^{-1}A_{1}^{T}W_{1}h_{1}$	9(4N−2)	
$W_{2}$	$B_{2}\mathrm{cov}(\psi_{1})$	729	2187
	$B_{2}\mathrm{cov}(\psi_{1})B_{2}^{T}$	729	
	${\left(B_{2}\mathrm{cov}(\psi_{1})B_{2}^{T}\right)}^{-1}$	729	
$\psi_{2}$	$A_{2}^{T}W_{2}$	567	1855
	$A_{2}^{T}W_{2}A_{2}$	441	
	${\left(A_{2}^{T}W_{2}A_{2}\right)}^{-1}$	343	
	${\left(A_{2}^{T}W_{2}A_{2}\right)}^{-1}A_{2}^{T}W_{2}$	441	
	${\left(A_{2}^{T}W_{2}A_{2}\right)}^{-1}A_{2}^{T}W_{2}h_{2}$	63	

**Table 3:** Estimated RMSE of the TS-WLS algorithm and the algorithm in this paper.

Estimated Parameters	Position		Velocity		$c^{o}$	
$\sigma^{2}$ (dB)	−5	20	−5	20	−5	20
TS-WLS (dB)	4.12	10.08	2.08	5.05	6.27	12.87
Proposed (dB)	0.66	6.48	2.06	4.99	5.10	11.39

## Data Availability

Data are contained within the article.

## Appendix A

In (12), the expression for each partial derivative is:

(A1) $$\begin{array}{l} \frac{\partial \gamma^{o}}{\partial \varphi }={\left[{\left(\frac{\partial \theta^{o}}{\partial \varphi }\right)}^{T},\;(\frac{\partial \beta^{o}}{\partial \varphi }{)}^{T},\;(\frac{\partial \tau^{o}}{\partial \varphi }{)}^{T},\;(\frac{\partial {\dot{\tau }}^{o}}{\partial \varphi }{)}^{T}\right]}_{(4N-2)\times 6}^{T}\\ \frac{\partial \gamma^{o}}{\partial c^{o}}={\left[{\left(\frac{\partial \theta^{o}}{\partial c^{o}}\right)}^{T},\;(\frac{\partial \beta^{o}}{\partial c^{o}}{)}^{T},\;(\frac{\partial \tau^{o}}{\partial c^{o}}{)}^{T},\;(\frac{\partial {\dot{\tau }}^{o}}{\partial c^{o}}{)}^{T}\right]}_{(4N-2)\times 1}^{T}\\ \frac{\partial \gamma^{o}}{\partial S^{o}}={\left[{\left(\frac{\partial \theta^{o}}{\partial S^{o}}\right)}^{T},\;(\frac{\partial \beta^{o}}{\partial S^{o}}{)}^{T},\;(\frac{\partial \tau^{o}}{\partial S^{o}}{)}^{T},\;(\frac{\partial {\dot{\tau }}^{o}}{\partial S^{o}}{)}^{T}\right]}_{(4N-2)\times 6N}^{T} \end{array}$$

Based on (2), (4), (6), and (A1), it can be calculated as follows:

1. $\frac{\partial \theta^{o}}{\partial \varphi },\frac{\partial \beta^{o}}{\partial \varphi }$ are the N×6 matrices with their $n_{th}$ row matrices, respectively:

(A2) $$\frac{\partial \theta^{o}}{\partial \varphi }(n,:)=p_{n}^{T}/\left(R_{n}^{o}\cos\left(\beta_{n}^{o}\right)\right)$$

where $p_{n}={\left[-\sin\left(\theta_{n}^{o}\right),\cos\left(\theta_{n}^{o}\right),O_{1\times 4}\right]}^{T}$.

(A3) $$\frac{\partial \beta^{o}}{\partial \varphi }(n,:)=q_{n}^{T}/R_{n}^{o}$$

where $q_{n}^{T}={\left[-\sin\left(\beta_{n}^{o}\right)\cos\left(\theta_{n}^{o}\right),-\sin\left(\beta_{n}^{o}\right)\sin\left(\theta_{n}^{o}\right),\cos\left(\beta_{n}^{o}\right),O_{1\times 3}\right]}^{T}$.

2. $\frac{\partial \tau^{o}}{\partial \varphi },\;\frac{\partial {\dot{\tau }}^{o}}{\partial \varphi }$ are the (N−1)×6 matrices with their ${(n-1)}_{th}$ row matrices, respectively:

(A4) $$\frac{\partial \tau^{o}}{\partial \varphi }(n-1,:)=\left[\left(\rho_{u^{o},s_{n}^{o}}^{T}-\rho_{u^{o},s_{1}^{o}}^{T}\right)/c^{o},O_{1\times 3}\right]$$

where $\rho_{a,b}=(a-b)/\|a-b\|$.

(A5) $$\frac{\partial {\dot{\tau }}^{o}}{\partial \varphi }(n-1,:)=\left[q_{u,s_{n}^{o}}^{T}-q_{u,s_{1}^{o}}^{T},\rho_{u^{o},s_{n}^{o}}^{T}-\rho_{u^{o},s_{1}^{o}}^{T}\right]/c^{o}$$

where $q_{a,b}=\left(I_{3}-\left(\frac{(a-b){\left(a-b\right)}^{T}}{\|a-b{\|}^{2}}\right)\right)\frac{\left(\dot{a}-\dot{b}\right)}{\left\|a-b\right\|}$, $I_{3}$ is a 3×3 unit matrix.

(A6) $$\frac{\partial {\dot{\tau }}^{o}}{\partial u^{T}}(n-1,:)=\left[\left(\frac{{\left({\dot{u}}^{o}-{\dot{s}}_{n}^{o}\right)}^{T}}{R_{n}^{o}}-\frac{{\left(u^{o}-s_{n}^{o}\right)}^{T}{\dot{R}}_{n}^{o}}{{\left(R_{n}^{o}\right)}^{2}}\right)-\left(\frac{{\left({\dot{u}}^{o}-{\dot{s}}_{1}^{o}\right)}^{T}}{R_{1}^{o}}-\frac{{\left(u^{o}-s_{1}^{o}\right)}^{T}{\dot{R}}_{1}^{o}}{{\left(R_{1}^{o}\right)}^{2}}\right)\right]$$

(A7) $$\frac{\partial {\dot{\tau }}^{o}}{\partial u^{T}}(n-1,:)=\left[\frac{{\left({\dot{u}}^{o}-{\dot{s}}_{n}^{o}\right)}^{T}}{R_{n}^{o}}\left(I_{3}-\frac{\left(u^{o}-s_{n}^{o}\right){\left(u^{o}-s_{n}^{o}\right)}^{T}}{{\left(R_{n}^{o}\right)}^{2}}\right)-\frac{{\left({\dot{u}}^{o}-{\dot{s}}_{1}^{o}\right)}^{T}}{R_{1}^{o}}\left(I_{3}-\frac{\left(u^{o}-s_{1}^{o}\right){\left(u^{o}-s_{1}^{o}\right)}^{T}}{{\left(R_{1}^{o}\right)}^{2}}\right)\right]$$

3. $\frac{\partial \theta^{o}}{\partial c^{o}},\;\frac{\partial \beta^{o}}{\partial c^{o}}$ are N×1 zero matrices. $\;\frac{\partial \tau^{o}}{\partial c^{o}},\;\frac{\partial {\dot{\tau }}^{o}}{\partial c^{o}}$ are (N−1)×6 matrices with their ${(n-1)}_{th}$ row matrices, respectively:

(A8)
$$\begin{array}{l} \frac{\partial \tau^{o}}{\partial c}\left(n-1,:\right)=-\left(R_{n}^{o}-R_{1}^{o}\right)/c^{o2}\\ \frac{\partial {\dot{\tau }}^{o}}{\partial c^{o}}\left(n-1,:\right)=-\left({\dot{R}}_{n}^{o}-{\dot{R}}_{1}^{o}\right)/c^{o2} \end{array}$$

4. $\frac{\partial \theta^{o}}{\partial S^{o}},\;\frac{\partial \beta^{o}}{\partial S^{o}}$ are N×6N matrices with their $n_{th}$ row matrices, respectively:

(A9)
$$\begin{array}{l} \frac{\partial \theta^{o}}{\partial S^{o}}\left(n,:\right)=-\left[O_{1\times 3(n-1)},\frac{\partial \theta^{o}}{\partial \varphi }(n,:),O_{1\times (6N-3n)}\right]\\ \frac{\partial \beta^{o}}{\partial S^{o}}\left(n,:\right)=-\left[O_{1\times 3(n-1)},\frac{\partial \beta^{o}}{\partial \varphi }(n,:),O_{\left(6N-3n\right)}\right] \end{array}$$

5. $\frac{\partial \tau^{o}}{\partial S^{o}},\;\frac{\partial {\dot{\tau }}^{o}}{\partial S^{o}}$ are (N−1)×6N matrices with their ${(n-1)}_{th}$ row matrices, respectively:

(A10)
$$\begin{array}{l} \frac{\partial \tau^{o}}{\partial S^{o}}\left(n-1,:\right)=\left[\rho_{u^{o},s_{1}^{o}}^{T},O_{1\times 3(n-2)},-\rho_{u^{o},s_{n}^{o}}^{T},O_{1\times (6N-3n)}\right]/c^{o}\\ \frac{\partial {\dot{\tau }}^{o}}{\partial S^{o}}\left(n-1,:\right)=\left[q_{u,s_{1}^{o}}^{T},O_{1\times 3(n-2)},-q_{u,s_{n}^{o}}^{T},O_{1\times 3(N-n)},\rho_{u^{o},s_{1}^{o}}^{T},O_{1\times 3(N-2)},-\rho_{u^{o},s_{n}^{o}}^{T},O_{1\times 3(N-n)}\right]/c^{o} \end{array}$$

## Appendix B

Since $G_{1}=B_{1}^{-1}A_{1}B_{2}^{-1}A_{2}B_{3}^{-1}$, taking the individual matrices and multiplying them, the individual matrices can be obtained:

(A11) $$G_{1}\left(n,:\right)=\left[\frac{-\sin\left(\theta_{n}\right)}{R_{n}\cos(\beta_{n})},\frac{\cos\left(\theta_{n}\right)}{R_{n}\cos(\beta_{n})},O_{1\times 5}\right]$$

(A12) $$G_{1}\left(n+N,:\right)=\left[\frac{-\sin\left(\beta_{n}\right)\cos\left(\theta_{n}\right)}{R_{n}},\frac{-\sin\left(\beta_{n}\right)\sin\left(\theta_{n}\right)}{R_{n}},\frac{\cos(\beta_{n})}{R_{n}},O_{1\times 4}\right]$$

(A13) $$G_{1}\left(n+2N-1,1:6\right)=-\left[\frac{{\left(s_{n}-s_{1}\right)}^{T}}{R_{n}c^{o}}+\frac{\tau_{n}\Psi_{1}(7){\left(\Psi_{1}(1:3)-s_{1}\right)}^{T}}{R_{n}c^{o}\Psi_{1}(8)},O_{1\times 3}\right]$$

(A14) $$G_{1}\left(n+2N-1,7\right)=-\left[\frac{\tau_{n}^{2}}{R_{n}}-\frac{\tau_{n}{\left\|\Psi_{1}(1:3)-s_{1}\right\|}^{2}}{R_{n}\Psi_{1}(8)}\right]$$

(A15) $$\begin{array}{ll} G_{1}\left(n+3N-2,1:3\right) & =\left[\frac{{\dot{R}}_{n}{\left(s_{n}-s_{1}\right)}^{T}}{R_{n}^{2}c^{o}}\right.-\frac{{\left(s_{n}-s_{1}\right)}^{T}}{R_{n}c^{o}}+\frac{\Psi_{1}(7)}{\Psi_{1}(8)}\left(\frac{\tau_{n}{\dot{R}}_{n}}{R_{n}^{2}c^{o}}-\frac{{\dot{\tau }}_{n}}{R_{n}c^{o}}\right){\left(\Psi_{1}(1:3)-s_{1}\right)}^{T}\\ & \left.-\frac{{\dot{\tau }}_{n}}{R_{n}c^{o}}\left(\frac{\Psi_{1}(7)}{\Psi_{1}(8)}{\left(\Psi_{1}(4:6)-{\dot{s}}_{1}\right)}^{T}\right)+\frac{\Psi_{1}(7)\Psi_{1}(9)}{\Psi_{1}^{2}(8)}{\left(\Psi_{1}(1:3)-s_{1}\right)}^{T}\right] \end{array}$$

(A16) $$G_{1}\left(n+2N-1,4:6\right)=G_{1}\left(n+N-1,1:3\right)$$

(A17) $$\begin{array}{ll} G_{1}\left(n+2N-1,7\right)= & \left[\frac{2\tau_{n}}{R_{n}}\left(\frac{\tau_{n}{\dot{R}}_{n}}{2R_{n}}-{\dot{\tau }}_{n}-\frac{{\left(\Psi_{1}(1:3)-s_{1}\right)}^{T}(\Psi_{1}(4:6)-{\dot{s}}_{1})}{\Psi_{1}(8)}\right)\right.\\ & \left.\left(\frac{\tau_{n}{\dot{R}}_{n}}{R_{n}^{2}}-\frac{{\dot{\tau }}_{n}}{R_{n}}+\frac{\tau_{n}\Psi_{1}(9)}{R_{n}\Psi_{1}(8)}\right)\frac{{\left\|\Psi_{1}(1:3)-s_{1}\right\|}^{2}}{\Psi_{1}(8)}\right] \end{array}$$

Similarly, $G_{2}$ can be calculated as:

(A18) $$G_{2}\left(n,:\right)=\left[O_{1\times 3(n-1)},\omega_{1}{\left(\theta_{n}\right)}^{T}/R_{n}\cos(\beta_{n}),O_{1\times (6N-3n)}\right]$$

(A19) $$G_{2}\left(n+N,:\right)=\left[O_{1\times 3(n-1)},{\omega_{2}}^{T}\left(\theta_{n},\beta_{n}\right)/R_{n},O_{1\times (6N-3n)}\right],n=1,2\ldots ,N$$

(A20) $$G_{2}\left(n+2N-1,:\right)=-\left[\frac{{\left(u^{o}-s_{1}\right)}^{T}}{R_{1}c^{o}},O_{1\times 3(n-2)},-\frac{{\left(u^{o}-s_{n}\right)}^{T}}{R_{n}c^{o}},O_{1\times (6N-3n)}\right]$$

(A21) $$\begin{array}{l} G_{2}\left(n+3N-2,1:3N\right)=\left[\frac{{\dot{R}}_{n}{\left(u^{o}-s_{1}\right)}^{T}}{R_{n}R_{1}c^{o}}-\frac{{\left({\dot{u}}^{o}-{\dot{s}}_{1}+c^{o}\tau_{n}q_{u^{o},s_{1}}+c^{o}{\dot{\tau }}_{n}\rho_{u^{o},s_{1}}\right)}^{T}}{R_{n}c^{o}},\right.\\ O_{1\times 3(n-2)},\left.\frac{{\left({\dot{u}}^{o}-{\dot{s}}_{n}\right)}^{T}}{R_{n}c^{o}}-\frac{{\dot{R}}_{n}{\left(u^{o}-s_{n}\right)}^{T}}{R_{n}^{2}c^{o}},O_{1\times 3(N-n)}\right] \end{array}$$

(A22) $$G_{2}\left(n+3N-2,3N+1:6N\right)=\left[-\frac{{\left(u^{o}-s_{1}\right)}^{T}}{R_{1}c^{o}},O_{1\times 3(n-2)},\frac{{\left(u^{o}-s_{n}\right)}^{T}}{R_{n}c^{o}},O_{1\times 3(N-n)}\right]$$

where the value of $\Psi_{1}$ can be updated after obtaining the value of $\Psi_{2}$ to obtain $\Psi_{1}\approx \Psi_{1}^{o}$. Under the condition of satisfying (62), it can be shown that $c^{o}\tau_{n}\approx R_{n},c^{o}{\dot{\tau }}_{n}\approx {\dot{R}}_{n}$; by comparing the approximation operation of (A11)–(A14) with the partial derivatives in Appendix A, we can obtain $G_{1}\approx \left[\frac{\partial \gamma^{o}}{\partial \varphi^{o}}\;\frac{\partial \gamma^{o}}{\partial c^{o}}\right],G_{2}\approx -\frac{\partial \gamma^{o}}{\partial S^{o}}$. Therefore, the localization solving algorithm proposed in this paper can approximate the CRLB performance.

## References

[1] Hao K., Xue Q., Li C., Yu K. (2020). A Hybrid Localization Algorithm Based on Doppler Shift and AOA for an Underwater Mobile Node. IEEE Access.

[2] Meng X., Li Y., Wu Z., Hong S., Chang S. (2023). A Semidefinite Relaxation Approach for Mobile Target Localization Based on TOA and Doppler Frequency Shift Measurements. IEEE Sens. J..

[3] Wang Y., Wu Y. (2017). An Efficient Semidefinite Relaxation Algorithm for Moving Source Localization Using TDOA and FDOA Measurements. IEEE Commun. Lett..

[4] (2018). EvoLogics, Underwater Acoustic LBL Positioning Systems. https://evologics.com.

[5] Lavars N. DARPA Program Plunges into Underwater Positioning System. https://newatlas.com/darpa-underwater-navigation/43472/.

[6] Thomas H.G. GIB Buoys: An Interface between Space and Depths of the Oceans. Proceedings of the 1998 Workshop on Autonomous Underwater Vehicles (Cat. No.98CH36290).

[7] Youngberg J.W. (1991). A Novel Method for Extending GPS to Underwater Applications. Navigation.

[8] Jia T., Ho K.C., Wang H., Shen X. (2020). Localization of a Moving Object With Sensors in Motion by Time Delays and Doppler Shifts. IEEE Trans. Signal Process..

[9] Jia T., Liu H., Ho K.C., Wang H. (2023). Mitigating Sensor Motion Effect for AOA and AOA-TOA Localizations in Underwater Environments. IEEE Trans. Wirel. Commun..

[10] Hao K., Yu K., Gong Z., Du X., Liu Y., Zhao L. (2020). An Enhanced AUV- Aided TDoA Localization Algorithm for Underwater Acoustic Sensor Networks. Mob. Netw. Appl..

[11] Zhou R., Chen J., Tan W., Cai C. (2022). Sensor Selection for Optimal Target Localization with 3-D Angle of Arrival Estimation in Underwater Wireless Sensor Networks. J. Mar. Sci. Eng..

[12] Liu Y., Wang Y., Chen C., Liu C. (2023). Underwater Wireless Sensor Network-Based Localization Method under Mixed Line-of-Sight/Non-Line-of-Sight Conditions. J. Mar. Sci. Eng..

[13] Xiong Y., Wu N., Shen Y., Win M.Z. (2022). Cooperative Localization in Massive Networks. IEEE Trans. Inf. Theory.

[14] Ferreira B., Matos A., Cruz N. Optimal Positioning of Autonomous Marine Vehicles for Underwater Acoustic Source Localization Using TOA Measurements. Proceedings of the 2013 IEEE International Underwater Technology Symposium (UT).

[15] Zhang L., Zhang T., Shin H.-S. (2021). An Efficient Constrained Weighted Least Squares Method With Bias Reduction for TDOA-Based Localization. IEEE Sens. J..

[16] Nguyen N.H., Dogancay K. (2018). Closed-Form Algebraic Solutions for 3-D Doppler-Only Source Localization. IEEE Trans. Wirel. Commun..

[17] Li P., Liu Y., Yan T., Yang S., Li R. (2024). An Underwater Source Localization Method Using Bearing Measurements. Sensors.

[18] L. N. Nguyen T., Shin Y. (2019). An Efficient RSS Localization for Underwater Wireless Sensor Networks. Sensors.

[19] Zhang L., Zhang T., Shin H.-S., Xu X. (2021). Efficient Underwater Acoustical Localization Method Based On Time Difference and Bearing Measurements. IEEE Trans. Instrum. Meas..

[20] Pei Y., Zhang M., Guo F., Liu Q. Semidefinite Programming Approach for Source Localization Using TDOA, FDOA and AOA Measurements. Proceedings of the 2021 IEEE International Conference on Signal Processing, Communications and Computing (ICSPCC).

[21] Jiang F., Zhang Z. (2021). An Improved Underwater TDOA/AOA Joint Localisation Algorithm. IET Commun..

[22] Zheng Z., Zhang H., Wang W.-Q., So H.C. (2019). Source Localization Using TDOA and FDOA Measurements Based on Semidefinite Programming and Reformulation Linearization. J. Frankl. Inst..

[23] Kim J. (2023). Underwater Transmitter Localization Based on TDOA and FDOA Considering the Unknown Time-Varying Emission Frequency. J. Mar. Sci. Eng..

[24] Ho K.C., Xu W. (2004). An Accurate Algebraic Solution for Moving Source Location Using TDOA and FDOA Measurements. IEEE Trans. Signal Process..

[25] Ho K.C., Lu X., Kovavisaruch L. (2007). Source Localization Using TDOA and FDOA Measurements in the Presence of Receiver Location Errors: Analysis and Solution. IEEE Trans. Signal Process..

[26] Zhang F., Sun Y., Zou J., Zhang D., Wan Q. (2020). Closed-Form Localization Method for Moving Target in Passive Multistatic Radar Network. IEEE Sens. J..

[27] Kay S.M. (1993). Fundamentals of Statistical Signal Processing.

[28] Liu Y., Wang Y., Chen C. (2023). Efficient Underwater Acoustical Localization Method Based on TDOA with Sensor Position Errors. J. Mar. Sci. Eng..

[29] Diamant R., Lampe L. (2013). Underwater Localization with Time-Synchronization and Propagation Speed Uncertainties. IEEE Trans. Mob. Comput..

[30] Ramezani H., Jamali-Rad H., Leus G. (2013). Target Localization and Tracking for an Isogradient Sound Speed Profile. IEEE Trans. Signal Process..

[31] Yang S., Wang G., Ho K.C. (2021). Noise Resilient Solution and Its Analysis for Multistatic Localization Using Differential Arrival Times. Signal Process..

[32] Rui L., Ho K. (2015). Efficient Closed-Form Estimators for Multistatic Sonar Localization. IEEE Trans. Aerosp. Electron. Syst..

[33] Fan C., Wang D., Yang B., Yin J. (2022). An Algorithm for Underwater Target Localization of Multistatic Sonar System with Unknown Signal Propagation Speed. Acta Armamentarii.

[34] Jia T., Shen X., Wang H. (2019). Multistatic Sonar Localization With a Transmitter. IEEE Access.

[35] Sun T., Wang W., Chen P., Gao J.-J. (2023). Multistatic Localization Algorithm Under Unknown Signal Propagation Speed Scenario with Clock Synchronization and Sensor Position Errors. Acta Electron. Sin..

[36] Zhang B., Hu Y., Wang H., Zhuang Z. (2018). Underwater Source Localization Using TDOA and FDOA Measurements With Unknown Propagation Speed and Sensor Parameter Errors. IEEE Access.

[37] Zhang X.-D. (2017). Matrix Analysis and Applications.

[38] Grant M., Boyd S. CVX: Matlab Software for Disciplined Convex Programming. https://cvxr.com/cvx/.

